# Design and verification of an interlocked centrifugal exhaust system for hatch-type boiling disinfectors

**DOI:** 10.1371/journal.pone.0360909

**Published:** 2026-10-16

**Authors:** Xiaoxue Sun, Qinqin Wang, Wei Zheng

**Affiliations:** 1 Central Sterile Supply Department, Sichuan GEM Flower Hospital, North Sichuan Medical College, Chengdu, China; 2 Human Resources Department, Sichuan Integrative Medicine Hospital, Chengdu, China; 3 Central Sterile Supply Department, Sichuan Clinical Research Center for Cancer, Sichuan Cancer Hospital & Institute, Sichuan Cancer Center, Affiliated Cancer Hospital of University of Electronic Science and Technology of China, Chengdu, China; Johnson & Johnson, UNITED STATES OF AMERICA

## Abstract

**Objective:**

To address the occupational exposure risks caused by instantaneous steam overflow when opening the top cover of hatch-type boiling disinfectors in the central sterile supply department, this study developed an interlocked centrifugal exhaust system (ICES) characterized by operational simplicity and high adaptability.

**Methods:**

The ICES was assembled using a centrifugal fan, Hall effect switch, instant relay, time-delay relay, solid-state relay, and piping. The system achieved automatic start/stop of the fan based on the open‑or‑closed status of the top cover. A self-controlled design was adopted on one hatch-type boiling disinfector. Temperature was measured at the chamber edge (T1) and at the technician’s facial position (T2). Airborne particle counts were also measured at the technician’s facial position. Measurements were performed under conditions with and without the ICES enabled. Data were analyzed using the Wilcoxon signed-rank test.

**Results:**

After enabling the ICES, T1 and T2 decreased by 20.89% and 31.31%, respectively. The median temperature at T2 was 21.5 °C, which was close to the ambient temperature. The reduction ratios for airborne particle counts of six different sizes at the technician’s facial position ranged from 53.09% to 67.83%. All differences between groups were statistically significant (all P < 0.001).

**Conclusion:**

The ICES effectively reduces steam overflow upon the top-cover opening of hatch-type boiling disinfectors and lowers the risk of occupational injury. With advantages such as wide installation adaptability, imperceptible operation, and convenient maintenance, it provides a stable and reliable solution for the safety retrofitting of existing hatch-type boiling disinfectors.

## Introduction

The central sterile supply department (CSSD) is the department within a hospital responsible for the cleaning, disinfection, and sterilization of reusable medical devices, playing a pivotal role in controlling hospital-acquired infections [1–3]. Cleaning operations remove visible contaminants on medical devices; disinfection kills pathogenic microorganisms; and sterilization reduces the probability of a single viable microorganism remaining on a medical device to less than 10^-6^, thereby achieving the sterility assurance level. All three stages—cleaning, disinfection, and sterilization—are essential during the reprocessing of medical devices [4–8]. Disinfection methods are mainly classified into two categories: physical disinfection (moist heat, dry heat, ultraviolet, filtration, etc. [9–12]) and chemical disinfection (alcohol, chlorine dioxide, chlorine-containing disinfectants, peroxides, glutaraldehyde, quaternary ammonium salts, etc. [13–17]). Moist heat disinfection is utilized for heat‑and‑moisture‑resistant medical devices. It relies on the synergistic effect of moisture and high temperature to cause irreversible protein denaturation and coagulation, enzyme inactivation, cell membrane destruction, and nucleic acid damage, thereby blocking microbial metabolism and reproduction to eliminate pathogenic microorganisms [18,19]. Due to its advantages of high efficacy, no chemical residues, operational simplicity, and low cost, moist heat disinfection has become the primary disinfection method in the CSSD [12,20]. Moist heat disinfection and moist heat sterilization both utilize moisture and thermal energy to inactivate bacteria and viruses, yet they differ fundamentally in governing standards, microbial inactivation capacity, and clinical application scenarios, and must not be conflated. In accordance with the international organization for standardization (ISO) 15883 series, moist heat disinfection employs hot water as the medium, which only eliminates vegetative bacteria, viruses and some bacterial spores. Devices processed by this method fail to meet sterility requirements and are mainly used for pre-reprocessing of reusable instruments or disinfection of low-risk devices. By contrast, moist heat sterilization complies with ISO 17665, relying on saturated high-temperature and high-pressure steam inside a sealed chamber to achieve sterilization with a sterility assurance level (SAL) < 10^−6^, capable of completely eradicating all viable microorganisms including bacterial spores [21,22].

In CSSDs, moist heat disinfection operations are performed using automated washer-disinfectors or boiling disinfectors. Automated washer-disinfectors include single-chamber and multi-chamber configurations (washing chamber, ultrasonic chamber, disinfection chamber, and drying chamber), which can sequentially complete the cleaning, disinfection, and drying of medical devices. Moist heat disinfection is performed via hot water circulation spraying [23,24]. Boiling disinfectors exist only in a single-chamber configuration and are capable only of disinfection, which is achieved by immersing medical devices in high-temperature water. Within China’s medical device regulatory framework, boiling disinfectors are intended for moist heat disinfection of heat- and moisture-resistant medical devices and are classified as Class II medical devices. Pre-market registration testing is mandatory prior to commercial launch, while manufacturing and performance testing must strictly comply with the pharmaceutical industry standard YY/T (designation for Chinese pharmaceutical‑sector industry standards) 1687–2019. Each unit is delivered with complete manufacturer’s instructions for use that clearly specify all validated disinfection parameters, satisfying the clinical access requirements of CSSDs [25,26].

Certain precision micro-instruments, lumened devices and structurally complex instruments are incompatible with spray-based cleaning in automated washer-disinfectors and require manual cleaning followed by disinfection in boiling disinfectors. Precision micro-instruments are tiny or fragile, and are prone to loss or damage under the impact of high-flow spray from automated equipment; lumened and irregular complex devices contain numerous blind zones unreachable by pressurized water jets, preventing thorough internal cleaning. Automated washer-disinfectors cannot meet the cleaning demands of these special instruments, making boiling disinfectors a cost-effective supplementary alternative for CSSDs. Meanwhile, due to the low procurement cost, short disinfection operation time, and flexible operation of boiling disinfectors, they are widely used in many medical institutions, especially community hospitals.

The structure of a boiling disinfector includes a chamber (containing a heating element), a top cover, an outer shell, a control system, and water supply/drainage systems. Based on the opening mechanism of the top cover, boiling disinfectors are classified into two types: hatch-type (opening from front to back) and lift-type. In accordance with the requirements specified in ISO 15883 and YY/T 1687 for all medical moist heat disinfection equipment, healthcare facilities must complete a full set of three qualification procedures before boiling disinfectors are formally put into clinical use: installation qualification (IQ), operational qualification (OQ), and performance qualification (PQ) [21,25]. IQ verifies the matching of water and power utilities, original factory calibration certificates, and on-site installation conditions; OQ tests core performance metrics including heating rate, temperature control accuracy, timing function, and chamber door sealing under no-load conditions, and PQ validates stable attainment of target disinfection levels with standard clinical instrument loads, with annual re-qualification mandatory to ensure full compliance and controllability of the entire disinfection workflow.

The efficacy of moist heat disinfection is quantified by the A₀ value, defined as the equivalent time (in seconds) required to achieve identical microbial inactivation efficacy at a reference temperature of 80 °C. For medical devices scheduled for subsequent sterilization, the required A₀ value is 600 (90 °C for 1 min); for medical devices intended for direct clinical use after disinfection, the A₀ value shall reach 3000 (90 °C for 5 min, or 93 °C for 2.5 min) [4,19,21,27]. The operating procedure of a boiling disinfector is as follows. Water is first added to the designated water level line, the top cover is closed, and the unit is heated to the preset disinfection temperature. When disinfection is required, the top cover is opened, and cleaned medical devices are placed into the chamber and fully submerged. The top cover is then closed, and the timer is started. After 1–5 minutes, the top cover is opened, and the disinfected devices are retrieved [28].

During operation, the boiling disinfector continuously heats, accelerating water evaporation inside the chamber. As the top cover remains closed, the chamber is sealed, preventing the escape of steam. This leads to the accumulation of significant steam in the upper part of the chamber, creating a positive pressure environment. When the top cover is opened after disinfection, the accumulated steam rapidly surges into the indoor space. This poses a scalding risk for technicians and causes rapid fogging of protective eyewear or face shields, severely impairing vision and compromising both personal safety and workflow efficiency [29–31]. These occupational exposure risks are more pronounced with hatch-type boiling disinfectors. On the one hand, opening the top cover requires the technician to reach out and pull the handle, which forces the technician to stay in close proximity to the disinfector. With the top cover moving approximately 60 cm from front to back, the entire arm becomes fully exposed above the chamber, and the horizontal distance between the technician’s face and the chamber is also very short. As a result, the risks of scalding and visual obstruction are significantly increased. On the other hand, the manual opening of the top cover is typically completed within 1 second, causing the accumulated steam inside the chamber to be released in an even shorter timeframe, thereby increasing the risk of injury to the technician. In addition, the steam overflowing when the top cover is opened raises the temperature, humidity, and pressure in the CSSD decontamination area. The aerosols contained therein also heighten the risk of environmental and personnel infection [32].

Current solutions to the occupational hazards caused by steam overflow during the opening of boiling disinfectors primarily include passive protective measures, such as improved personal protective equipment (e.g., wearing anti-fog face shields), enhanced personal protection, and modified operating procedures (e.g., fully opening the hatch-type top cover from the side) [33,34]. These methods fail to reduce the total volume of overflowing steam at the source, offer limited protection, and rely on operator compliance, thereby presenting safety hazards due to potential human negligence. Some patents have proposed drilling holes in the top cover or chamber side and embedding a negative pressure suction system to continuously extract steam; others have proposed installing a wind deflector equipped with a negative pressure suction device above the disinfector. However, these patents remained at the design stage, and no products have been put into use. Furthermore, the described methods involved modifications to the main equipment body, requiring custom stainless steel components and internal piping. The difficulty of such modifications makes retrofitting or adding these systems to existing boiling disinfectors in CSSDs challenging, thus limiting their practical significance [35–37].

Therefore, it is urgent to address safety hazards induced by steam overflow, particularly for hatch‑type boiling disinfectors. This study aimed to answer the following research question: How can we effectively and safely reduce steam‑related occupational injury risks during top‑cover‑opening operations of hatch‑type boiling disinfectors? To answer this question, this study overcame the drawbacks of conventional passive protection strategies and developed an interlocked centrifugal exhaust system (ICES) featuring easy operation and broad adaptability. Experimental verification demonstrated that the ICES could stably mitigate steam‑associated occupational risks, which provided a practical technical solution for safety retrofitting of existing boiling disinfectors.

## Materials and methods

### Design and fabrication

The main components of the ICES included a centrifugal fan, a Hall effect switch, an instant relay, a time-delay relay, a solid-state relay, and suction/exhaust piping (Fig 1). The centrifugal fan operated at 220 V, with a power of 750 W, a rotational speed of 2800 r/min, and an air volume of 1200 m^3^/h. It was made of stainless steel, featuring an inlet diameter of 150 mm and an outlet diameter of 100 mm. The Hall effect switch (Model HLD-20NC) provided a negative-positive-negative (NPN) output in normally closed mode and operated at 12 V. Both the instant relay (low-level trigger) and the time-delay relay required a 12 V direct current input. The time-delay relay was programmed for delay-off after power-on, with an adjustable delay ranging from 0 to 5 minutes; it disconnected immediately if power was interrupted during the delay period. The solid-state relay (Model DA 40A) had an input voltage of 12 V, a load voltage range of 24–480 V, and a load current of 40 A. The suction pipeline was made of polyvinyl chloride (PVC) with a diameter of 150 mm, fitted with a rectangular acrylonitrile butadiene styrene (ABS) suction inlet (250 mm × 80 mm) at the front end, while the exhaust pipeline consisted of aluminum corrugated hose.

**Fig 1:**
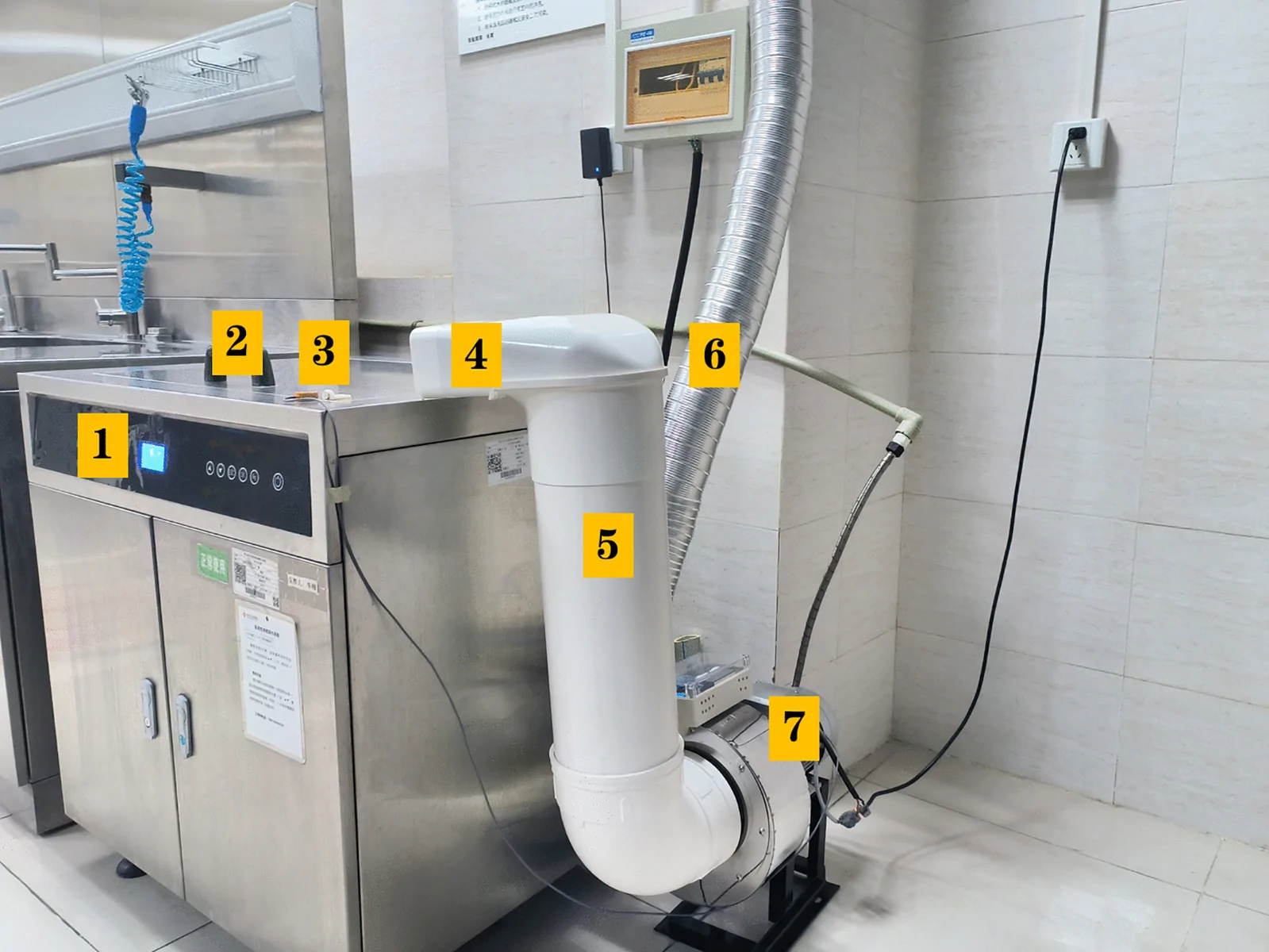
Interlocked Centrifugal Exhaust System. 1. Boiling disinfector; 2. Top cover and handle; 3. Hall switch and magnet; 4. Rectangular suction inlet; 5. Suction pipe; 6. Exhaust pipe; 7. Centrifugal fan.

All components were installed on one side of the boiling disinfector without altering the original structure. A circular magnet was vertically adhered to the edge of the top cover. The sensing surface of the Hall switch was fixed to the chamber edge, facing the magnet at a distance of less than 10 mm. The lower edge of the rectangular suction inlet was secured flush against the side edge of the chamber and connected to the centrifugal fan inlet via PVC piping; the fan outlet was connected to the CSSD main exhaust duct via aluminum corrugated hose. The centrifugal fan was mounted on a metal bracket and connected to a 220 V power supply. A separate 12 V power supply simultaneously powered the Hall switch, instant relay, time-delay relay, and solid-state relay. The signal line of the Hall switch was connected to the interface A of the instant relay; the normally open (NO) output interface of the instant relay was connected to the positive input of the time-delay relay; the NO interface of the time-delay relay was connected to the positive input of the solid-state relay. The two output interfaces of the solid-state relay were connected to any one power line of the centrifugal fan (Fig 2). All relays were housed in an ABS waterproof junction box, with several circular holes drilled in the side walls for heat dissipation.

**Fig 2:**
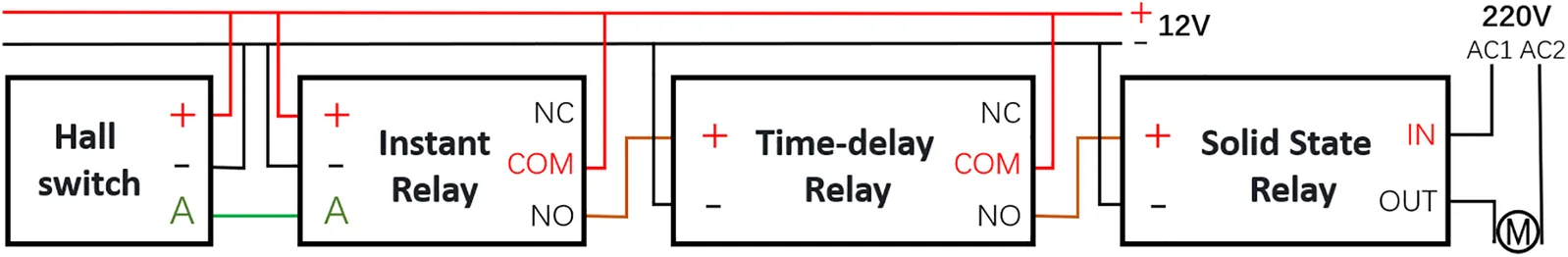
Wiring diagram of the interlocked centrifugal exhaust system. DC 12V and AC 220V were electrically isolated. DC positive (red), negative (black), and signal line (green). M represents the centrifugal fan.

The ICES achieved automatic start/stop based on the open‑or‑closed status of the boiling disinfector’s top cover, requiring no manual intervention. When the top cover was closed, the magnet faced the sensing area of the Hall switch, which continuously output a high-level signal. In this state, the instant relay did not work, and the ICES remained in standby mode. The moment the top cover began to open, the magnet left the sensing area, causing the Hall switch to flip and output a low-level signal. This engaged the instant relay, which sequentially triggered the time-delay relay and solid-state relay to conduct, thereby starting the centrifugal fan. The fan rapidly extracted overflowing steam from the chamber, preventing scalding of technicians and fogging of face shields. Based on actual operational scenarios, the risk of steam overflow predominantly existed within the first minute after top cover opening. Therefore, the ICES was programmed with a power-on delay auto-shutoff logic: the centrifugal fan automatically stopped after running for 1 minute, avoiding energy consumption from ineffective idling. If the top cover was closed early during the fan’s delay cycle, the Hall switch re-sensed the magnetic signal, and the ICES immediately cut off the output, stopping the fan. This enabled on-demand start/stop operation. The timing control logic of the ICES is illustrated in Fig 3. The ICES operated with imperceptible linkage throughout, without altering the original operating procedures or increasing the workload of technicians.

**Fig 3:**
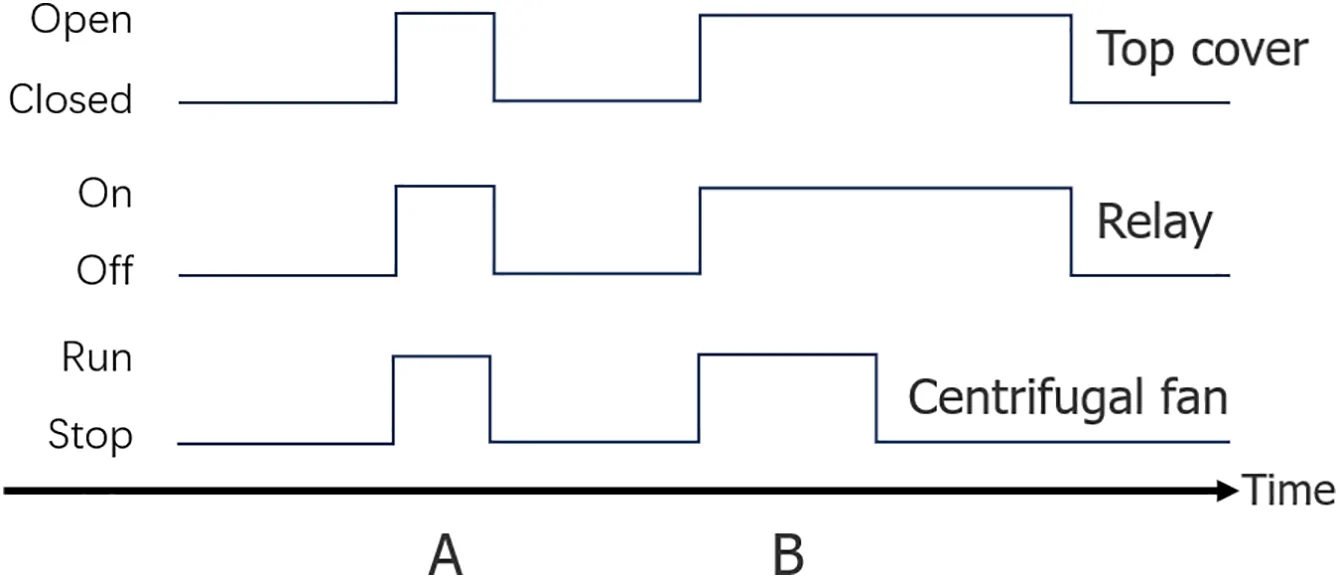
Timing diagram of the interlocked centrifugal exhaust system. When the top cover opened, the Hall switch triggered the relay action, starting the centrifugal fan. If the cover closed within 1 minute (A), the fan stopped synchronously with the cover; if the cover remained open for more than 1 minute (B), the fan continued to run until automatically stopping after 1 minute.

### Effectiveness verification

Since CSSD technicians faced greater occupational injury risks when using hatch-type boiling disinfectors, one hatch-type disinfector was selected to verify the application effect of the ICES. This disinfector was sourced from the CSSD of a general hospital with 600 beds (Brand: Shandong Xinhua; External dimensions: 800 mm × 640 mm × 830 mm; Chamber volume: 80 L; Water volume during operation: 60 L). The disinfector had passed full IQ, OQ and PQ validation, and all built-in temperature sensors, heating modules and timing controllers of the unit underwent annual metrological calibration in strict accordance with the manufacturer’s instructions and YY/T 1687 standard. The test site was the CSSD decontamination area, with a temperature of 19 ± 2 °C and relative humidity of 50 ± 5%. To reduce interfering factors, the disinfector operated under no-load conditions, meaning that no medical devices were placed inside during testing. No human participants or human biological samples were involved in this experimental study. This validation was a preliminary prototype test performed on a single hatch-type boiling disinfector.

Manual opening presented issues such as inconsistent opening speeds and angles, which directly affected the speed and range of steam overflow, causing high dispersion in experimental data. To unify experimental conditions and control variables, a self-made uniform-speed top‑cover-opening auxiliary device was used to standardize the top cover opening speed. A heavy-duty hook was adhered to the wall above the rear of the disinfector, with a fixed pulley installed on the hook. A rope passed through the pulley and was fixed to the top cover handle. Simultaneously, a 50 cm ruler was vertically adhered to the wall, with distinct marks made every 10 cm (Fig 4). During testing, a uniform pulling method was adopted: a technician pulled the rope downward at a speed of 10 cm per second, completing the top cover opening operation over 5 consecutive seconds. The opening angles of the top cover were 30°, 45°, 60°, 73°, and 86°, respectively. The opening angle was limited to less than 90°, since the traction rope could not provide sufficient structural support; the top cover would tip backward and collapse under gravity if the angle exceeded 90°.

**Fig 4:**
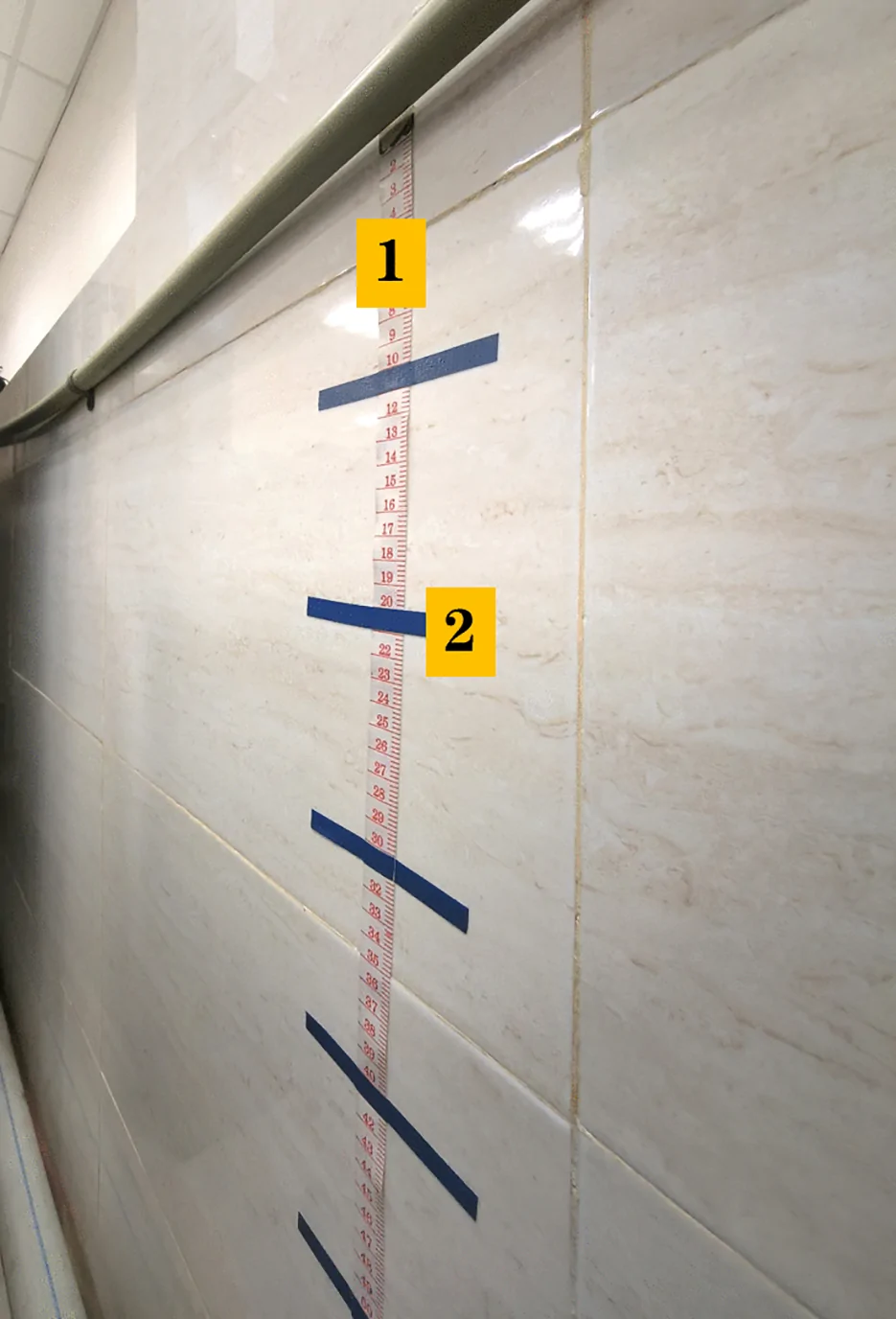
Wall positioning ruler for controlling top cover opening speed. A ruler (1) was vertically adhered to the wall behind the boiling disinfector, with distinct marks (2) every 10 cm. During the experiment, the tester pulled the rope downward at 10 cm/s to achieve standardized control of the top cover opening of the boiling disinfector.

The experiment utilized a dual-channel thermometer and an airborne particle counter as testing tools, both of which had been calibrated by accredited metrological institutions. The dual-channel thermometer (Model UT 320D, Uni-Trend Technology (China) Co., Ltd., China) could measure temperatures at two locations simultaneously with a resolution of 0.1 °C. The airborne particle counter (Model MKS 800, Tianjin Maikasi Technology Co., Ltd., China) had a working flow rate of 1 L/min and could simultaneously measure particle counts for six sizes: 0.3 μm, 0.5 μm, 1.0 μm, 2.5 μm, 5.0 μm, and 10 μm. Temperature probe T1 (chamber edge) was fixed at the center of the sink edge on the front of the disinfector (1 cm from the stainless steel to reduce metal interference). Temperature probe T2 and the particle counter sampling port were fixed at a height of 1.5 m at the center front of the disinfector (simulating the technician’s facial position), as shown in Fig 5. This study adopted a self-controlled design. The condition without the ICES served as the control group. The boiling disinfector was set to run under no-load conditions at 93 °C for 3 minutes before opening the top cover. The maximum values of T1, T2, and particles of different sizes within 1 minute were recorded, with 20 repeated measurements. When using the ICES, data were collected using the same method. Subsequently, time-series temperature monitoring was additionally performed, in which T1 and T2 temperatures were recorded once per second over 0–15 s after top cover opening for both the control and ICES groups. The monitoring duration was set to 15 s based on our preliminary observations: regardless of whether the ICES was activated, temperatures at both measurement sites returned to baseline levels within 15 s after top cover opening. Therefore, extending the monitoring period beyond 15 s would not provide additional information on the effective exposure window.

**Fig 5:**
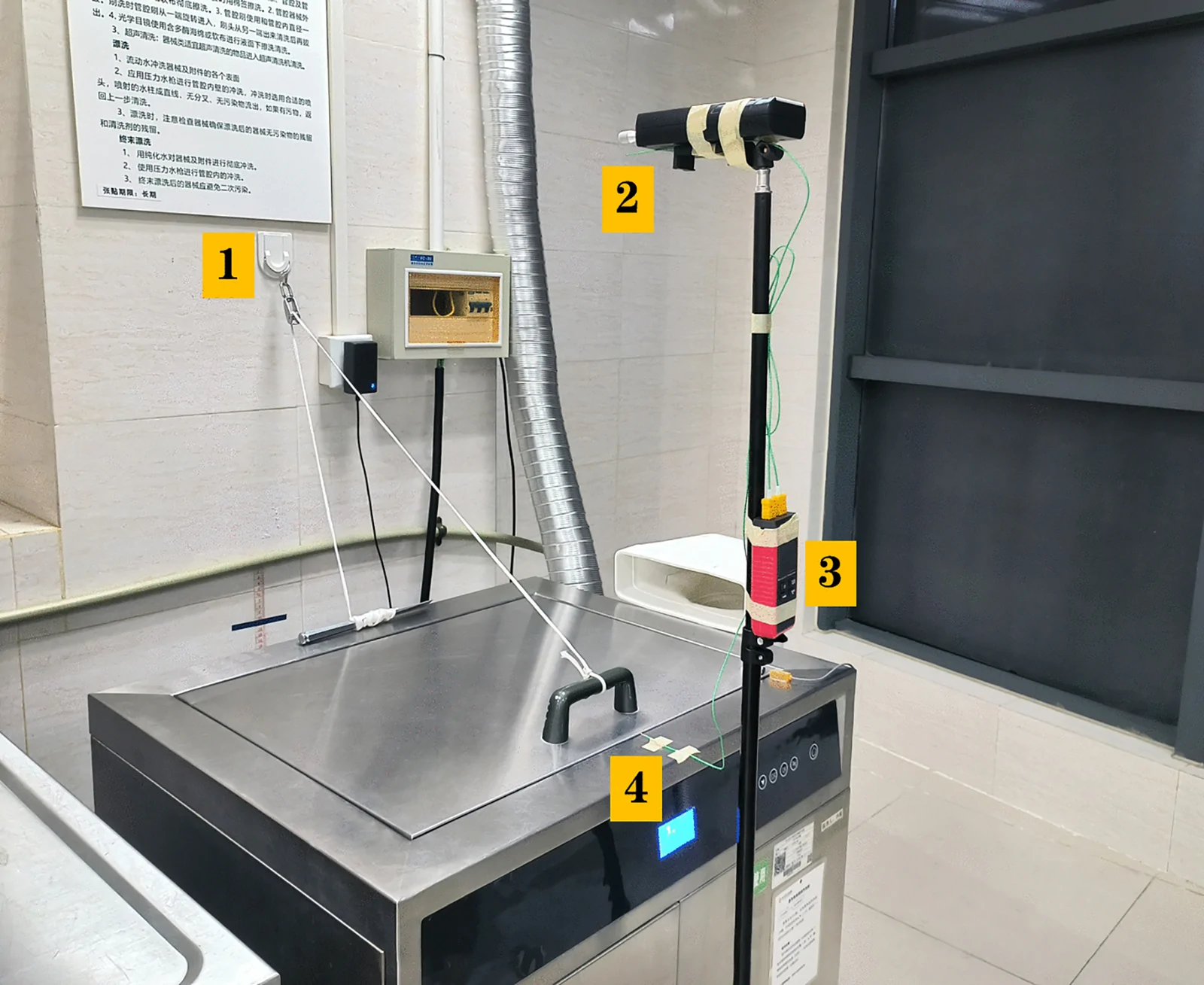
Layout of experimental measurement points. 1. Hook, fixed pulley, and traction rope; 2. Airborne particle counter and temperature (T2) sampling point; 3. Dual-channel thermometer; 4. Temperature (T1) sampling point.

### Statistical methods

R software (version 4.5.1) was used for data analysis. All measurement data were first subjected to normality tests. As some data did not conform to a normal distribution, all measurement data were expressed as M (P_25_, P_75_), and inter-group comparisons were conducted using the Wilcoxon signed-rank test. A P-value < 0.05 was considered statistically significant.

## Results

The temperatures (T1 and T2) and airborne particle counts (0.3 μm, 0.5 μm, 1.0 μm, 2.5 μm, 5.0 μm, and 10 μm) in the ICES group were significantly lower than those in the control group, decreasing by 20.89%, 31.31%, 53.09%, 65.22%, 66.91%, 67.10%, 67.83%, and 67.65%, respectively (calculated as median‑based reduction). All P-values were < 0.001, indicating statistically significant differences (Table 1).

**Table 1:** Comparison of temperature and airborne particle counts between the two groups (n = 20, M (P_25_, P_75_)).

Groups	Temperature		Airborne particulate counts					
	T1	T2	0.3 μm	0.5 μm	1.0 μm	2.5 μm	5.0 μm	10 μm
Control Group	38.3 (37.4, 42.8)	31.3 (30.0, 32.5)	663001 (638245, 689631)	364924 (345929, 394475)	79056 (73981, 86264)	8300 (7754, 9054)	7641 (7043, 8318)	1113 (1046, 1210)
ICES Group	30.3 (29.0, 31.0)	21.5 (20.3, 21.9)	311002 (293071, 328006)	126926 (116953, 149829)	26158 (24072, 30559)	2731 (2277, 3194)	2458 (2213, 2874)	360 (286, 416)
V-value	210	210	210	210	210	210	210	210
P-value	< 0.001	< 0.001	< 0.001	< 0.001	< 0.001	< 0.001	< 0.001	< 0.001

Based on the temperature data collected at 1-second intervals for the control and ICES groups over 0–15 s after top cover opening, time-series temperature curves were plotted (Fig 6). In the control group, the temperatures at the chamber edge (T1) and the technician’s facial position (T2) rose rapidly immediately upon top cover opening, creating a distinct high-temperature exposure window. After activating the ICES, the temperatures at both measurement sites showed almost no noticeable elevation throughout the entire top-cover-opening process, and the transient high-temperature exposure window induced by steam was nearly eliminated.

**Fig 6:**
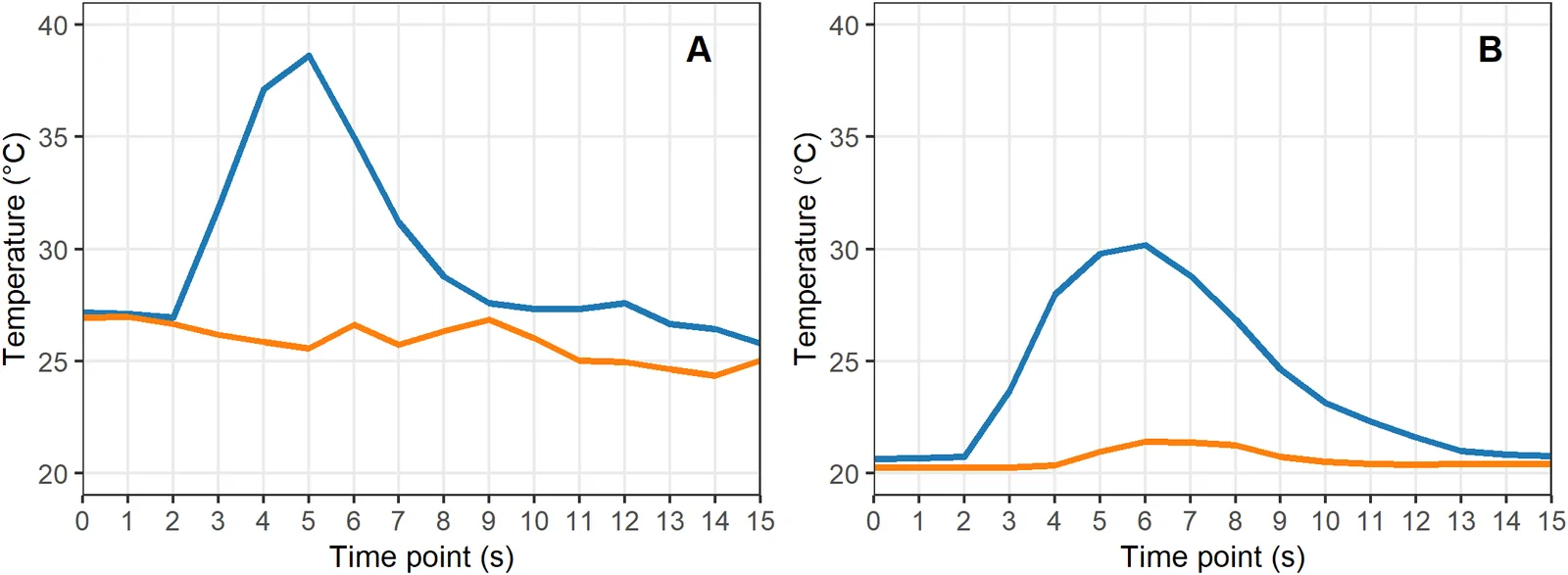
Time-series temperature profiles of T1 and T2 within 0-15 s after top cover opening for the control and ICES groups. Blue curves: control group (ICES disabled); orange curves: ICES group (ICES activated). Subplot A shows T1 at the chamber edge; Subplot B shows T2 at the technician’s facial position.

## Discussion

### Preliminary testing showed the ICES significantly reduced occupational injury risks from steam overflow in hatch-type boiling disinfectors

The risk of rapid steam overflow upon top cover opening is inherently a design limitation of hatch-type boiling disinfectors, rather than a consequence of insufficient equipment performance, improper installation or non-compliant operating procedures. The tested unit fully complied with factory and hospital quality control requirements, and all operational protocols were strictly followed throughout the experiment. Instant steam surging occurs immediately after rapid top cover opening as a physical outcome of positive pressure accumulation within the sealed chamber, which cannot be eliminated by standardized disinfection routines or conventional ventilation adjustments. In line with the engineering control priority principle specified in ISO 14971 medical device risk management, external auxiliary exhaust assemblies can be adopted as an engineering control measure to mitigate such inherent occupational hazards that cannot be fully eliminated by routine operations or personal protective equipment, which serves as the core design rationale for developing the ICES system in this study [38].

The overflow of steam when opening a boiling disinfector is difficult to quantify directly and precisely; therefore, this study used a combination of temperature detection and airborne particle counting for comprehensive evaluation [39,40]. All efficacy verification tests of the ICES were conducted under no-load conditions for two objective reasons. First, all precision micro-instruments and lumened devices of the test hospital were stored in the operating room warehouse for standby use after reprocessing, and no spare instruments could be allocated for experimental loading. Second, all medical devices are fully submerged beneath the water surface throughout boiling disinfection, with saturated steam accumulating in the headspace above the liquid level; there are no significant differences in chamber temperature or total steam generation during operation whether instrument loads are placed inside the chamber or not. In the self-controlled experiment, after enabling the ICES, the temperatures at the chamber edge (T1) and the technician’s facial position (T2) decreased by 20.89% and 31.31%, respectively. Notably, the median temperature at T2 was 21.5 °C, which was basically consistent with the ambient experimental temperature. Simultaneously, the ICES significantly reduced the level of airborne suspended particulate matter in the operating area, with the reduction ratio for six different particle sizes at the technician’s facial position ranging from 53.09% to 67.83%.

Analyzing the working principle, the ICES relied on a millisecond-level sensing linkage strategy to trigger the centrifugal fan instantly upon the opening of the disinfector’s top cover. The rectangular suction inlet, positioned tightly against the upper edge of the chamber, precisely corresponded to the steam overflow gap formed during the initial opening. Leveraging the high negative pressure suction capacity of the centrifugal fan, it rapidly extracted the instantaneously overflowing steam. Without the ICES, the overflowing steam diffused vertically upward as a mass of white fog, directly impacting the technician’s arm and facial work areas. With the ICES enabled, the overflowing steam was rapidly extracted by the instantaneous negative pressure, with no obvious white fog rising at the scene. This study also conducted an additional fogging test to further corroborate this conclusion: when nine CSSD technicians used the hatch-type disinfector equipped with the ICES, no fogging occurred on their glasses or face shields. The quantitative data and intuitive experimental phenomena jointly confirmed that, under the tested no-load conditions in this single-unit preliminary test, the ICES could efficiently extract the large volume of steam overflowing at the moment of opening, improving the operating environment and effectively reducing the risk of occupational injury to technicians.

### Advantages and promotion value of the ICES

The ICES exerts no potential adverse impacts on the process performance and disinfection efficacy of boiling disinfectors. A small magnet and a Hall sensor are only adhesively mounted on the edges of the top cover and chamber, without altering the original chamber structure, heating circuits, temperature control system, water pipeline system and door sealing assemblies of the disinfector. The control circuit of the ICES and the power supply for the centrifugal fan are completely electrically isolated from the boiling disinfector, eliminating the need for any rewiring or hardware disassembly of the original medical device. This non-intrusive external installation means that the ICES retrofit will not interfere with the core thermal disinfection cycle, temperature accuracy, chamber pressure, or disinfection performance under loaded conditions of the disinfector, nor will it compromise any validated critical process parameters.

The ICES provides fully automated operation with no increased workload for staff. The ICES achieved automatic start/stop via hardware linkage logic, activating the centrifugal fan the moment the top cover began to open, without requiring manual operation of the fan switch. This “imperceptible operation” mode did not alter the standard operating procedures for instrument loading, disinfection, and retrieval, incurred no additional learning costs, and did not increase the workload of technicians, resulting in high acceptance among them.

The ICES has a simple structure and low maintenance costs. The ICES did not employ complex programmable control systems but used conventional hardware such as relays, Hall switches, and fans for physical assembly, eliminating software failures at the source and enhancing operational stability. The modular design meant components worked independently; any damaged part could be disassembled and replaced individually, simplifying maintenance. Except for the centrifugal fan, other sensing and control components were inexpensive, keeping overall costs controllable. Furthermore, the fan was made of stainless steel, piping was constructed from PVC and stainless steel, and relays were housed in ABS waterproof boxes, allowing the ICES to withstand the high-temperature and humid CSSD environment. The smooth outer surface was easy to clean, meeting hospital infection control requirements [41,42]. In terms of operational stability, the ICES prototype ran continuously in the decontamination area of the CSSD for four consecutive months. No faults or attenuation of sensing sensitivity were observed in the centrifugal fan, Hall sensor and time-delay control modules, and no issues such as circuit short-circuiting or pipeline deformation occurred, which preliminarily verified its reliability for short-term continuous operation.

The ICES offers strong scenario adaptability. As an external accessory, the ICES was added to the disinfector without destructive modifications such as disassembly. Even if removed, the disinfector could be restored to its original state. The installation positions of the fan and Hall switch were flexible, and the piping was highly malleable. Combined with its non-destructive installation feature, the ICES was less constrained by installation space and could be well adapted to different types of boiling disinfectors in CSSDs.

The dual‑shutdown logic balances protection, energy‑saving performance, and noise reduction. The fan automatically stopped after a 1-minute delay, based on the premise that steam overflow volume was very low after 1 minute, avoiding energy waste. If the top cover was closed early within that minute, the device sensed the magnetic signal and stopped instantly, avoiding idling. This on-demand start-stop function reduced power consumption and lowered noise while ensuring protection.

Additionally, boiling disinfectors are classified into hatch-type and lift-type. For lift-type boiling disinfectors, when the top cover is opened, steam overflow channels exist around the cover, and the rising speed of the cover is slow (>5 s), which effectively avoids the phenomenon of steam surging upward instantaneously. Simultaneously, the top cover rises automatically after disinfection, requiring no close-proximity manual operation by technicians. These advantages result in a very low risk of occupational injury from steam overflow for lift-type boiling disinfectors. Therefore, CSSDs should prioritize lift-type boiling disinfectors within their budgets when purchasing boiling disinfectors in the future to avoid occupational exposure risks at the hardware source. The ICES developed in this study was primarily designed for safety upgrades of existing hatch-type boiling disinfectors.

### Limitations

This study had certain limitations requiring further improvement:

Limitations in sample representativeness and testing methods. This study tested only one hatch-type boiling disinfector, and the limited sample size might affect representativeness and generalizability of the results. Additionally, the evaluation method was relatively simple, using only temperature and particle counting at the technician’s facial position, failing to fully reflect environmental changes in the surrounding area. Future research will include multi-dimensional systematic testing on more models of boiling disinfectors to evaluate the performance of the ICES more comprehensively and accurately.

High operating noise of the ICES. The maximum operating noise of the centrifugal fan reached 80 dB, causing auditory interference to technicians. The typical ambient noise level within CSSD work zones ranges from 60–65 dB. Since the fan runs for no more than 1 minute per activation, the duration of high-noise exposure is limited, and associated adverse impacts are expected to be mild. Nevertheless, formal evaluation of operator comfort should be conducted in future field trials. Future plans include selecting quieter centrifugal fans and appropriately reducing fan power if necessary to lower operating noise, balancing steam protection with workplace comfort.

Condensate residue in the volute. Due to condensation of water vapor upon contact with cold surfaces, a small amount of condensate remained inside the volute after the ICES’s operation. Although this did not affect current functionality, it posed a potential hygiene risk during long-term operation. A 4-mm drainage port at the bottom of the fan volute connected to a plastic drainage tube was bench-tested and demonstrated effective condensate removal. Subsequent prototype iterations will adopt this design to achieve automatic condensate discharge, further improving the sanitary safety and maintenance convenience of the device.

## Conclusion

The ICES effectively reduced the amount of steam overflowing into the operating environment when opening boiling disinfectors. With advantages such as wide installation adaptability, imperceptible operation, and convenient maintenance, it provided a simple and efficient solution for mitigating the risk of occupational injury caused by steam from hatch-type boiling disinfectors.

## Supporting information

S1 Data Experimental_Test_Data. (XLSX)
