# Supplementary material for: Design and verification of an interlocked centrifugal exhaust system for hatch-type boiling disinfectors
Source: PLoS One. 2026 Oct 16;21(10):e0360909. doi: 10.1371/journal.pone.0360909 (PMC13674762; doi:10.1371/journal.pone.0360909)
Supplement: Supplementary file 1 [file pone.0360909.s003.docx]

Response to Reviewers

We sincerely appreciate the constructive comments raised by the academic editor and reviewers. Revisions and supplements have been made to the manuscript as suggested. Detailed point-by-point responses are provided below.

Response to academic Editor

Comment 1. Insufficient description and regulatory context of the "boiling disinfector"

Response: To address the inadequate description of equipment information and regulatory background of boiling disinfectors, the following revisions have been implemented:

1. Supplementary information on equipment classification and market compliance. In the Introduction section, we clarify that boiling disinfectors fall under Class II medical devices in China, complying with the industrial standard YY/T 1687-2019. Each device is delivered with complete manufacturer Instructions for Use (IFU) that specify standardized validated disinfection parameters. Full descriptions of the statutory classification, intended clinical applications, and market access requirements of the equipment are provided.

2. Improved framework of mandatory equipment qualification. Combined with ISO 15883 and YY/T 1687 standards, we elaborate on three mandatory qualification procedures (IQ, OQ, PQ) that all medical moist heat disinfection equipment must complete before clinical deployment, illustrate the inspection scope of each qualification stage, and supplement comprehensive content regarding standardized quality control of the equipment.

3. Supplementary calibration qualification of the tested unit. The boiling disinfector used in all tests passed full IQ/OQ/PQ validation. All built-in temperature sensors, heating assemblies and timing controllers received annual metrological calibration in accordance with manufacturer IFU and YY/T 1687-2019. The equipment operated under fully compliant conditions throughout the experiment, eliminating data bias caused by equipment drift or parameter deviation.

4. Demonstration that ICES retrofitting does not invalidate original equipment qualification status. A standalone paragraph has been added to the Discussion section to illustrate that ICES is an external non-destructive auxiliary exhaust assembly. Only adhesive-mounted magnets and Hall sensors are installed without drilling, disassembly or modification of original wiring. No alterations are made to the chamber structure, heating system, temperature control program, water pipeline, door sealing or other core process modules, so the original IQ/OQ/PQ validated status of the boiling disinfector remains intact.

5. Clarification of the root source of occupational risks. The Discussion section states that steam overflow during hatch opening is not induced by defective equipment, improper installation or non-compliant operations. Instead, it represents an inherent physical design limitation of front-flip hatch-type boiling disinfectors. This clear differentiation of risk origins excludes insufficient equipment quality control as a contributing factor.

Comment 2. Clarification of disinfection versus sterilization principles

Response:

1. Distinct differentiation between moist heat disinfection and moist heat sterilization. In the Introduction section, we distinguish the two thermal processing modalities across four dimensions (heat transfer medium, microbial inactivation capacity, sterility assurance level, and clinical application) based on two international standards: ISO 15883 and ISO 17665.

2. Unified supporting standards for equipment processing. YY/T 1687-2019 is specified as the exclusive industrial standard for boiling disinfectors throughout the manuscript. The A₀ value evaluation criterion is cross-referenced with ISO 15883 and the domestic CSSD standard WS 310.3, clearly defining standardized disinfection temperatures, holding durations and benchmarks for microbial inactivation.

3. Elaborated the important clinical application scenarios of boiling disinfectors. We expand descriptions of the clinical demand for precision micro-instruments and lumened devices, explain the limitations of automated spray washer-disinfectors including instrument loss and unreachable cleaning dead zones, fully illustrate the clinical necessity of boiling disinfectors, and clarify their applicable scenarios.

Comment 3. Lack of standards-based discussion

Response: To remedy the insufficient standard-related discussion in the original manuscript, we have integrated multiple sets of standards covering moist heat disinfection, medical device quality control, risk management and equipment qualification throughout the text:

1. Standards for moist heat disinfection and equipment qualification. Frequent citations of ISO 15883, YY/T 1687 and WS 310.3 are incorporated to fully interpret the mandatory industrial requirements for A₀ value assessment and IQ/OQ/PQ validation of thermal disinfection equipment.

2. Medical device risk management standards. ISO 14971 risk management guidelines are cited in the root-cause risk analysis paragraph. Following the risk control hierarchy of "engineering control priority", we demonstrate that the external ICES exhaust assembly constitutes a compliant engineering intervention to mitigate inherent occupational hazards of the equipment, filling the gap in risk management standard discussion.

3. Verification of ICES compliance with equipment regulatory requirements. The manuscript clarifies that ICES is a non-intrusive external add-on solution without destructive modification or adjustment of core process parameters of the boiling disinfector, which will not compromise the original validated equipment status and conforms to regulatory specifications governing medical device modification risk control.

Comment 4. Root cause analysis of occupational risk is missing

Response: To address the absence of comprehensive root-cause analysis for occupational exposure hazards, a full analytical paragraph has been added at the start of the Discussion section to decompose risk contributors layer by layer:

1. The tested boiling disinfector completed full IQ/OQ/PQ validation. Its hardware configuration, installation layout and routine maintenance fully comply with manufacturer IFU and industrial standards, ruling out inadequate equipment performance, defective installation or incomplete quality control as risk drivers.

2. All experimental operations strictly followed standardized manufacturer protocols with standardized operator movements, eliminating excessive steam overflow caused by non-compliant manual handling.

3. Instant massive steam surge upon hatch opening is an objective physical phenomenon arising from positive pressure accumulation within the sealed heated chamber. The front-flip hatch design forces technicians to stand in close proximity to high-temperature steam plumes, which cannot be eliminated by standardized disinfection cycles or basic room ventilation alone.

4. In line with ISO 14971 risk management logic, steam overflow hazards are inherent structural limitations of the equipment as manufactured. Personal protective equipment and procedural optimization cannot fully eliminate such risks, which makes external engineering control devices such as ICES a targeted and necessary risk mitigation strategy.

Comment 5. Limited verification of effectiveness

Response: We objectively acknowledge the experimental limitations and supplement engineering principles and short-term stability data to strengthen argumentation:

1. We acknowledge the limitations of this study. All validation tests were performed on only one hatch-type boiling disinfector. The limited sample size restricts the generalizability of the research conclusions to a certain extent. In follow-up studies, we will incorporate additional models of equipment for testing to enrich the experimental dataset. The ICES features a simple structure and operating principle. It realizes protective function via magnetic induction hatch detection and instantaneous negative-pressure steam extraction and requires no customized modifications for specific equipment models. Its modular external installation combined with flexibly bendable pipelines enables compatibility with devices of various shapes. Based on the design characteristics of the device, we anticipate that this system is applicable to most commercially available hatch-type boiling disinfectors.

2. All effectiveness verification tests were carried out under no-load conditions, for two objective reasons. Firstly, all precision micro-instruments and lumened devices at the testing hospital were reserved for standby use in operating theatres during the trial period, and no spare instruments were available for experimental loading. Secondly, all medical devices are fully submerged under the water surface throughout the boiling disinfection cycle; saturated steam accumulates in the headspace above the liquid level, and there is no remarkable difference in chamber temperature or total steam volume generated during operation regardless of whether instrument loads are placed inside the chamber.

3. Supplementary data on continuous operational reliability. Four months of continuous on-site clinical operation data are added to the Discussion section. The ICES prototype ran daily alongside routine boiling disinfection workflows, with zero failures of the centrifugal fan, Hall sensor and delay control modules, no attenuation of sensing sensitivity, and no circuit short-circuiting or pipeline deformation observed, which preliminarily verifies its short-term operational stability.

4. ICES exerts no adverse influence on the inherent performance of the host boiling disinfector, with a standalone explanatory paragraph added to the Discussion. ICES adopts a non-destructive external installation scheme that leaves all core performance metrics of the disinfector unchanged, including disinfection cycle duration, internal chamber temperature and A₀ value. This retrofitting solution does not raise additional maintenance costs, nor will it invalidate the original Installation Qualification, Operational Qualification and Performance Qualification status of the host equipment.

Comment 6. PLOS ONE reproducibility requirements

Response: To meet the journal’s reproducibility criteria, we supplemented standardized information regarding equipment, measuring instruments and retrofitting solutions to enable independent replication of the entire experiment:

1. Complete specifications and qualification records of the boiling disinfector. Full documentation of the tested unit’s brand, external dimensions, chamber volume and standard working water volume is retained in the manuscript. Supplementary text confirms full IQ/OQ/PQ validation and annual metrological calibration of all core components, with all experimental workflows conducted in strict accordance with ISO 15883 to ensure consistent, repeatable test conditions.

2. Supplementary calibration information for measuring instruments. We specify that both the dual-channel thermometer and airborne particle counter were sourced from certified manufacturers and calibrated by accredited metrological institutions to guarantee repeatable and comparable detection data.

3. Comprehensive hardware parameter documentation for ICES. The manuscript retains complete dimensional, material and electrical specifications of all components including the centrifugal fan, Hall effect switch, relays and piping, providing full hardware references for independent replication. The selected centrifugal fan provides sufficient airflow redundancy. Minor fluctuations in flow rate will not impair steam extraction efficiency. This study focuses on on-site performance verification rather than computational fluid dynamics simulation. Therefore, the supplied hardware parameters are adequate to replicate the system, and supplementary fan-sizing calculation is beyond the scope of the present work.

4. Enhanced risk assessment for equipment modification. The manuscript fully analyzes the non-destructive external installation, complete electrical isolation and zero alteration of core equipment processes of ICES.

Response to Reviewer

Comment 1:You are focusing almost exclusively on the occupational hazards but sterilization efficacy and not increasing the risk for recontamination are also important. Could you add a comment on whether you believe this setup might increase the recontamination risk, e.g. through the additional airflow? Sterilization efficacy often has to be validated (and routinely checked). Regarding the guidelines applicable to your institution, would the addition of the exhaust system require a revalidation of the machines? Does loading/unloading the disinfector change the expected temperature and/or particle distribution?

Response: First, the ICES serves as an independent external auxiliary device. Only a magnet and a Hall sensor are adhesively attached to the boiling disinfector, without any modifications to the disinfector’s chamber, temperature control system, water pipeline or sealing structure. Its installation does not alter any critical process parameters validated via IQ/OQ/PQ, and relevant discussions have been provided in the manuscript. In accordance with the Chinese standard YY/T 1687 and the hospital’s equipment management specifications, a full revalidation of the host disinfection equipment is not required for such an independent external auxiliary protection device.

Second, the system does not elevate risks of environmental contamination or recontamination of instruments. The core components of ICES include a stainless steel centrifugal fan and corrosion-resistant exhaust pipelines with smooth surfaces that resist microbial adhesion. The extracted steam is directly discharged outdoors through the building’s centralized exhaust duct system and will not aggravate contamination within the CSSD workspace.

Finally, instrument loading and unloading operations cannot change the temperature and aerosol distribution associated with instantaneous steam release upon hatch opening. As illustrated in the experimental design section of the manuscript, all instruments remain fully submerged under the water surface throughout boiling disinfection. Submerged instrument loads exert minimal influence on the total volume of saturated steam accumulated in the headspace above the liquid level.

Comment 2:Abstract: Please write the “p” in “p-value” in lower case.
Response: Following the reviewer’s suggestion, "P-value" in the Abstract has been revised to the lowercase form "p-value".

Comment 3: Introduction: Line 74 Considering the broad readership of PONE, it might be helpful to give a short description of the definition of the A0 Value (equivalent time (in seconds) at a reference temperature of 80 °C required to achieve the same microbial inactivation as the actual thermal process).
Response: We have supplemented the complete definition of the A₀ value at the corresponding position in the Introduction, consistent with standard formal definitions.

Comment 4: Lines 126 – 129 Considering the steam comes from a disinfector, why do you assume it to increase the “risk of environmental and personnel infection”? Could you provide literature evaluating the infection risk from disinfectors?
Response: Steam generated during boiling disinfection itself poses low infection risk. However, the decontamination area of the CSSD is classified as a contaminated zone. Massive hot steam surges outward immediately after hatch opening, disturbing pre-existing bioaerosols in the environment, facilitating aerosol suspension and uncontrolled dispersion in ambient air, and thereby elevating exposure risks for staff. Relevant literature citations have been added to this paragraph of the manuscript to support this viewpoint.

Comment 5: Methods: If you add a sentence to your experimental design emphasizing that you did not involve human participants, the supplementary file stating no humans were involved might not be necessary.
Response: We have added a statement in the manuscript clarifying that this trial involved no experiments on human participants and recruited no human subjects. Meanwhile, in compliance with PLOS ONE submission requirements, we retain the relevant declaration regarding the absence of human participants within the supplementary file to prevent format-related issues during manuscript evaluation.

Comment 6: Lines 236 – 242 If manual opening leaves the data unreliable, is there still an element of compliance involved, even with the exhaust, i.e. is there still a risk for people opening the disinfector rapidly?
Response: A distinction should be drawn between standardized experimental test conditions and routine clinical application scenarios. The self-made uniform-speed hatch-opening auxiliary device described in the manuscript was adopted solely to eliminate confounding variables induced by inconsistent manual hatch-opening speeds and ensure comparability of experimental datasets across groups; it was used exclusively for quantitative performance testing of the ICES.

When the ICES system is deployed in real clinical settings, this auxiliary device is not required. Regardless of variations in operators’ hatch-opening speed, the Hall sensor activates the exhaust fan immediately upon initial hatch opening to extract steam, thereby mitigating massive steam overflow and reducing risks of occupational exposure.

Comment 7: Text for Fig 4: Please explicitly explain numbers 1 and 2 shown in the image in the image description. Line 256 Please add the manufacturer of the thermometer. Line 258 Please add the manufacturer of the particle counter. Line 280 Please write the “p” in “p-value” in lower case.
Response: All revisions have been completed. Labels 1 and 2 in Figure 4 are explicitly defined within the figure legend. Manufacturer information for the dual-channel thermometer and airborne particle counter has been supplemented in the specified lines. All instances of "P-value" throughout the manuscript have been corrected to the lowercase form "p-value".

Comment 8: Results: I would suggest adding the data table to the main manuscript and publish the whole document without supplementary information. This would increase accessibility, and it does not seem to be too much data.

Response: After comprehensive consideration of manuscript length and layout of figures and tables, we prefer to keep the data tables as supplementary information. All supplementary materials are openly accessible without impairing data availability. The final formatting arrangement will follow subsequent instructions from the journal editors.
