# Supplementary material for: Design and verification of an interlocked centrifugal exhaust system for hatch-type boiling disinfectors
Source: PLoS One. 2026 Oct 16;21(10):e0360909. doi: 10.1371/journal.pone.0360909 (PMC13674762; doi:10.1371/journal.pone.0360909)
Supplement: Supplementary file 2 [file pone.0360909.s004.docx]

Response to Reviewers

We sincerely appreciate the reviewers’ constructive comments and thoughtful suggestions, which have substantially helped us improve the quality and clarity of this manuscript. We have carefully addressed each comment point‑by‑point as below.

Reviewer #2:

Comment 1. The title appears a little convoluted. It could be more precise and clearer.

Response: After careful evaluation, we prefer to retain the original title: *Design and verification of an interlocked centrifugal exhaust system for hatch‑type boiling disinfectors*. This title comprehensively covers all core elements of the present study, including the research activities (design and verification), the full name of the proposed interlocked centrifugal exhaust system (ICES), and the target equipment (hatch‑type boiling disinfectors). No ambiguous wording is used. All essential metadata required for literature indexing and database retrieval are included. Therefore, we keep the original title unchanged in the revised manuscript.

Comment 2. Provide the achieved results in the form of percentages in the last section of your abstract.

Response: We prepared the abstract following common writing practices for biomedical‑engineering research manuscripts. Our abstract consists of four distinct sections: objective, methods, results, and conclusion. All quantitative percentage‑based efficacy outcomes of ICES are fully and explicitly presented within the Results section of the Abstract. The Conclusion section is designed to interpret the practical implications and application value drawn from our experimental findings, instead of restating detailed numerical results, which would create unnecessary textual redundancy. Readers can retrieve all key percentage‑reduction data from the Results section of the Abstract. For these reasons, we keep the original wording of the Abstract Conclusion unchanged.

Comment 3. Provide the key contribution of your work in the form of a Research Question (RQ).

Response: Thank you for this suggestion. Following the narrative logic of the three recommended reference papers, we have re‑phrased the final paragraph of the Introduction. A research question (RQ) is explicitly stated at the beginning of this paragraph, which is immediately followed by the key outcomes and contributions of the present study. No new substantive content has been added; all information is inherited from the original manuscript, with only sentence structure and expression revised. This revision can be found in the final paragraph of the Introduction in the revised manuscript.

Comment 4. The abstract must be reorganized to clearly state the purpose, methodology, and key quantitative results. The introduction needs a logical flow that connects background, problem, gap, and motivation. The authors should follow the below papers and improve it accordingly.
i. Advanced federated ensemble internet of learning approach for cloud based medical healthcare monitoring system
ii. High-precision brain tumor classification from MRI images using an advanced hybrid deep learning method with minimal radiation exposure
iii. Deep learning-based multi-class classification of thyroid disorders on Tc-99m scintigraphy using modified DenseNet-201

Response: Thank you for this constructive suggestion and for providing the three reference papers. We have learned from their explicit narrative framework, and relevant adjustments have already been implemented for the formulation of the research question and study contributions as described in Comment 3.

For the abstract, the current version is structured sequentially with objective, methods, key quantitative results and conclusion sections, which systematically reports the research purpose of mitigating occupational exposure risks of hatch‑type boiling disinfectors, the configuration of the interlocked centrifugal exhaust system (ICES), experimental measurement indicators, statistical approaches, core quantitative reduction data of temperature and airborne particle counts, as well as the practical significance of this work. All required information elements are already fully covered within the existing abstract, and no further reorganization of the abstract structure is performed.

For the Introduction, the existing text follows the logical chain of background‑clinical problem‑research gap‑research motivation. It begins with the routine disinfection modalities adopted in the central sterile supply department, elaborates the widespread application scenario of moist‑heat disinfection, explains the indispensable supplementary role of boiling disinfectors for special complex instruments, describes the operating workflow of hatch‑type boiling disinfectors, and further illustrates the occupational exposure hazards induced by instantaneous steam overflow during top‑cover opening. Subsequently, the limitations of currently available protective strategies and patented technical solutions are comprehensively discussed to clarify the existing research gap. Finally, the research motivation of developing the ICES is presented. This complete logical progression meets the narrative requirement raised by you. Combined with the revision in Comment 3, an explicit research question together with key study outcomes has been added at the end of the Introduction to further strengthen the logical closure of this section. Considering that the core narrative and argumentation are already complete, we retain the original organizational structure of the Introduction without additional large‑scale rearrangement.

Comment 5. Don’t use abbreviations in the keywords.

Response: We have double‑checked our keywords. The keyword list was submitted via the journal submission system rather than being embedded within the main manuscript file. The submitted keywords are as follows: Central sterile supply department; Boiling disinfector; Occupational protection. All keywords are written in complete full‑length terms without any abbreviations.

Comment 6. At the end of the related works section provide a paragraph that should encapsulate on the knowledge that what shortcomings has motivated you to do this specific work.

Response: We understand that you are asking for a concise encapsulation of the shortcomings of existing approaches that motivated the present research. This content is fully presented in the penultimate paragraph of the Introduction. This paragraph systematically summarizes the limitations of passive protective measures and existing patented technical solutions for mitigating steam‑overflow‑related occupational hazards, and clarifies why these existing strategies cannot resolve the clinical challenges of hatch‑type boiling disinfectors. This paragraph serves exactly the summarizing function you requested. Adding another standalone summary paragraph would introduce redundant repetitive information without bringing new insights. Accordingly, we retain the original text of this paragraph without further revision. Together with the newly added research question and key outcomes in the final paragraph of the Introduction (see Comment 3), the logical chain from identified shortcomings to research motivation is complete.

Comment 7. Some abbreviations are not defined for their first appearance.

Response: We have systematically re‑examined every acronym and symbol throughout the abstract and full‑text manuscript. We have verified that each abbreviation is accompanied by its complete full name upon its first occurrence, including ICES, CSSD, ISO, SAL, IQ, OQ, PQ, PVC, ABS and A₀. Symbols T1 and T2 are also clearly defined in the Methods subsection of the Abstract before they are used in the Results subsection. No undefined abbreviations or symbols remain in the revised manuscript.

Comment 8. There are a lot of grammatical and typos; the authors are suggested to do a strong proofread before the revision submission and remove them.

Response: We have conducted comprehensive proof‑reading across the entire manuscript. Several typographical mistakes, duplicated words and awkward grammatical expressions were identified and corrected, including the duplicated definite article “the” and other minor wording imperfections.

Comment 9. Add a time-series measurement (e.g., temperature vs. time graphs for the first 30 seconds post-opening) to show if the ICES also shortens the exposure window.

Response: We greatly appreciate this suggestion. Supplementary experiments were carried out to record temperature variations at the two measurement sites with and without the ICES. The two positions were the chamber edge (T1) and the technician's facial position (T2). Time-series plots were generated based on the acquired data.

Preliminary tests revealed that temperatures at both sampling positions returned to baseline levels within 15 seconds after opening the top cover, regardless of whether the ICES was activated. For this reason, temperature monitoring was terminated at 15 s, and we did not perform measurements covering the 30 s after top cover opening.

The time-series plots clearly demonstrate that the occupational injury risk induced by high-temperature steam is substantially reduced when the ICES is activated. Specifically, after ICES deployment, temperatures at both positions show nearly no obvious rise upon opening the top cover, and the time window for steam exposure is almost eliminated. The new experimental data and figures have been integrated into the revised manuscript.

Comment 10. State clearly if the machine was tested multiple times on the same day or across days to account for environmental variables (e.g., ambient room temperature, humidity). If not randomized, add this as a limitation.

Response: All experiments were conducted across three non‑consecutive days. The test time points were not randomly selected. However, the environmental temperature and humidity within the central sterile supply department are maintained in a relatively stable and controlled range. In addition, the test equipment, measuring instruments, operators and objective test parameters remained consistent throughout all trials. Given these stable conditions, we consider the impact of non‑randomized test timing negligible. Therefore, we do not include this point in the Limitations section.

Comment 11. While not mandatory, adding a measurement of relative humidity or air velocity at T2 would provide a more complete risk assessment (thermal discomfort is a combination of heat and humidity).

Response: We apologize that local relative humidity and air velocity at T1 and T2 were not recorded during the experiments. As described in our response to Comment 10, the central sterile supply department is an enclosed space with strictly controlled temperature and humidity. Variations in local humidity and air velocity were minimal under these stable indoor conditions. In addition, high‑sensitivity instruments for point measurement of these parameters were unavailable, and on‑site measurement at these locations presented practical operational challenges. We believe that the absence of these two measurements does not affect the core conclusions of this study.

Reviewer #3:
First, the experimental limitations (testing on only one machine and under no‑load conditions) are disclosed only in the final Limitations section, while the Results and the opening of the Discussion use strong, generalised phrasing. I recommend stating these constraints explicitly in the Methods, for instance by adding a sentence that this is a preliminary single‑unit no‑load validation, and slightly moderating the wording in the Discussion to reflect the preliminary scope.
Response: We have added a sentence in the Methods section to state that this validation is a preliminary single-unit prototype test. In addition, we have revised the heading of the first Discussion subsection and moderated the opening statements of Discussion to clearly reflect the preliminary and limited scope of the present experiment, avoiding overgeneralized conclusions.

Second, the fan noise (80 dB) and condensate residue are mentioned but no plans or feasibility assessments are provided. I suggest adding brief comments: for noise, note that the ambient CSSD noise is typically 60‑65 dB and that operator comfort should be evaluated; for condensate, mention that a drainage port has been bench‑tested and will be included in future iterations. The authors’ previous revisions were exemplary, and these remaining adjustments are straightforward. I therefore recommend acceptance after minor revision.

Response: We appreciate this thoughtful suggestion. We have supplemented relevant discussions in the Limitations section. For fan noise, we added background CSSD ambient noise levels (60–65 dB) and noted that fan operation lasts less than one minute per use, which limits high-noise exposure. We also added that operator comfort assessment will be required in future field evaluations. For condensate residue, we added that a 4 mm drainage port with a plastic tube has completed bench testing with effective drainage performance, and this solution will be implemented in future prototype iterations.
