# Supplementary material for: Design and verification of an interlocked centrifugal exhaust system for hatch-type boiling disinfectors
Source: PLoS One. 2026 Oct 16;21(10):e0360909. doi: 10.1371/journal.pone.0360909 (PMC13674762; doi:10.1371/journal.pone.0360909)
Supplement: Peer review history

# Peer review: Decision Letter 0

**DOI:** 10.1371/journal.pone.0360909.r001

**Authors:** Paulo Laranjeira

15 Jul 2026

Dear Dr. Zheng,

**1. Insufficient description and regulatory context of the "boiling disinfector"**

A major limitation of the manuscript is the lack of information regarding the boiling disinfector itself. The authors describe the equipment as a device used for thermal disinfection in CSSDs, but provide limited information regarding its design, intended use, regulatory status, validation status, applicable standards, and manufacturer instructions for use (IFU).

Since medical devices reprocessed in healthcare facilities are expected to be processed according to validated manufacturer instructions and applicable standards, additional information is needed regarding:

- Device classification and intended use.
- Manufacturer specifications and validated operating parameters.
- Applicable national and international standards and regulations.
- Installation Qualification (IQ), Operational Qualification (OQ), and Performance Qualification (PQ) status of the equipment.
- Whether the proposed ICES modification affects the validated state of the equipment.

Without this information, it is difficult to determine whether the reported occupational risk originates from inherent limitations of the device design, improper installation, inadequate qualification, or operational practices rather than from the absence of the proposed ICES.

**2. Clarification of disinfection versus sterilization principles**

The manuscript repeatedly discusses moist heat and CSSD reprocessing but does not clearly distinguish between thermal disinfection and moist heat sterilization.

According to internationally recognized standards:

- Thermal disinfection is commonly achieved using hot water and characterized by achievement of a specified A0 value (e.g., ISO 15883 series).
- Moist heat sterilization is performed using saturated steam under controlled conditions and is governed by standards such as ISO 17665.

The term "boiling disinfector" is uncommon in current international CSSD practice and requires additional clarification. The authors should provide:

- A detailed technical description of the device and process.
- Operating temperatures and exposure times.
- Demonstration that the process achieves the intended level of microbial reduction.
- Applicable standards supporting use of this type of equipment in healthcare reprocessing environments.

Failure to clearly define the process raises concerns regarding reproducibility and interpretation of the results.

**3. Lack of standards-based discussion**

Although the manuscript proposes a safety retrofit for a device used in medical device reprocessing, there is no discussion of recognized standards governing:

- Thermal disinfection processes (e.g., ISO 15883 series).
- Reprocessing of reusable medical devices.
- Validation and qualification requirements.
- Manufacturer IFU compliance.
- Risk management principles for healthcare equipment.

The authors should explain how the proposed modification aligns with applicable standards and whether installation of the ICES alters the validated state of the original equipment.

**4. Root cause analysis of occupational risk is missing**

The manuscript assumes that steam release during opening is an unavoidable characteristic of hatch-type boiling disinfectors. However, no evidence is presented to determine whether the observed occupational exposure is caused by:

- Inherent equipment design limitations.
- Inadequate ventilation integration.
- Improper installation.
- Deficiencies in equipment qualification.
- Deviations from validated operating procedures.

A more comprehensive analysis is needed before concluding that a retrofit solution is the most appropriate or necessary mitigation strategy.

**5. Limited verification of effectiveness**

The study evaluates a single device under no-load conditions. While reductions in temperature and airborne particle counts are reported, important limitations remain:

- Only one disinfector model was evaluated.
- Testing was performed without representative medical device loads.
- Long-term operational reliability was not assessed.
- Potential effects on process performance, disinfection efficacy, equipment maintenance, and qualification status were not investigated.

The authors should clearly discuss these limitations and avoid overgeneralizing the applicability of the ICES to other equipment designs or healthcare settings.

**6. PLOS ONE reproducibility requirements**

PLOS ONE requires that experiments and methods be described in sufficient detail to enable independent reproduction of the work.

Additional information is needed regarding:

- The boiling disinfector design and specifications.
- Ventilation system characteristics.
- Validation and calibration of measurement systems.
- Engineering calculations supporting fan sizing and extraction performance.
- Risk assessment associated with modification of existing healthcare equipment.

These details are currently insufficient for independent replication of the proposed solution.

=============================

plosone@plos.org. When you're ready to submit your revision, log on to https://www.editorialmanager.com/pone/ and select the 'Submissions Needing Revision' folder to locate your manuscript file.

- A letter that responds to each point raised by the academic editor and reviewer(s). You should upload this letter as a separate file labeled 'Response to Reviewers'.
- A marked-up copy of your manuscript that highlights changes made to the original version. You should upload this as a separate file labeled 'Revised Manuscript with Track Changes'.
- An unmarked version of your revised paper without tracked changes. You should upload this as a separate file labeled 'Manuscript'.

If you would like to make changes to your financial disclosure, please include your updated statement in your cover letter. Guidelines for resubmitting your figure files are available below the reviewer comments at the end of this letter.

If applicable, we recommend that you deposit your laboratory protocols in protocols.io to enhance the reproducibility of your results. Protocols.io assigns your protocol its own identifier (DOI) so that it can be cited independently in the future. For instructions see: https://journals.plos.org/plosone/s/submission-guidelines#loc-laboratory-protocols. Additionally, PLOS ONE offers an option for publishing peer-reviewed Lab Protocol articles, which describe protocols hosted on protocols.io. Read more information on sharing protocols at https://plos.org/protocols?utm_medium=editorial-email&utm_source=authorletters&utm_campaign=protocols.

As the corresponding author, your ORCID iD is verified in the submission system and will appear in the published article. PLOS supports the use of ORCID, and we encourage all coauthors to register for an ORCID iD and use it as well. Please encourage your coauthors to verify their ORCID iD within the submission system before final acceptance, as unverified ORCID iDs will not appear in the published article. *Only*  the individual author can complete the verification step; PLOS staff *cannot*  verify ORCID iDs on behalf of authors.

We look forward to receiving your revised manuscript.

Kind regards,

Paulo Laranjeira

Academic Editor

PLOS One

Journal Requirements:

When submitting your revision, we need you to address these additional requirements.

1. Please ensure that your manuscript meets PLOS ONE’s style requirements, including those for file naming. The PLOS ONE style templates can be found at

https://journals.plos.org/plosone/s/file?id=wjVg/PLOSOne_formatting_sample_main_body.pdf and https://journals.plos.org/plosone/s/file?id=ba62/PLOSOne_formatting_sample_title_authors_affiliations.pdf

2. We note that Figure(s) 1, 4, 5 in your submission contain copyrighted images. All PLOS content is published under the Creative Commons Attribution License (CC BY 4.0), which means that the manuscript, images, and Supporting Information files will be freely available online, and any third party is permitted to access, download, copy, distribute, and use these materials in any way, even commercially, with proper attribution. For more information, see our copyright guidelines: http://journals.plos.org/plosone/s/licenses-and-copyright.

We require you to either (1) present written permission from the copyright holder to publish these figures specifically under the CC BY 4.0 license, or (2) remove the figures from your submission:

a. You may seek permission from the original copyright holder of Figure(s) 1, 4, 5 to publish the content specifically under the CC BY 4.0 license.

We recommend that you contact the original copyright holder with the Content Permission Form (http://journals.plos.org/plosone/s/file?id=7c09/content-permission-form.pdf) and the following text:

“I request permission for the open-access journal PLOS ONE to publish XXX under the Creative Commons Attribution License (CCAL) CC BY 4.0 (http://creativecommons.org/licenses/by/4.0/). Please be aware that this license allows unrestricted use and distribution, even commercially, by third parties. Please reply and provide explicit written permission to publish XXX under a CC BY license and complete the attached form.”

Please upload the completed Content Permission Form or other proof of granted permissions as an "Other" file with your submission.

In the figure caption of the copyrighted figure, please include the following text: “Reprinted from [ref] under a CC BY license, with permission from [name of publisher], original copyright [original copyright year].”

b. If you are unable to obtain permission from the original copyright holder to publish these figures under the CC BY 4.0 license or if the copyright holder’s requirements are incompatible with the CC BY 4.0 license, please either i) remove the figure or ii) supply a replacement figure that complies with the CC BY 4.0 license. Please check copyright information on all replacement figures and update the figure caption with source information. If applicable, please specify in the figure caption text when a figure is similar but not identical to the original image and is therefore for illustrative purposes only.

3. Please include captions for your Supporting Information files at the end of your manuscript, and update any in-text citations to match accordingly. Please see our Supporting Information guidelines for more information: http://journals.plos.org/plosone/s/supporting-information.

4. If the reviewer comments include a recommendation to cite specific previously published works, please review and evaluate these publications to determine whether they are relevant and should be cited. There is no requirement to cite these works unless the editor has indicated otherwise.

[Note: HTML markup is below. Please do not edit.]

Reviewers' comments:

Reviewer’s Responses to Questions

**Comments to the Author**

1. Is the manuscript technically sound, and do the data support the conclusions?

Reviewer #1: Yes

**********

2. Has the statistical analysis been performed appropriately and rigorously?

Reviewer #1: Yes

**********

3. Have the authors made all data underlying the findings in their manuscript fully available?

The PLOS Data policy

Reviewer #1: Yes

**********

4. Is the manuscript presented in an intelligible fashion and written in standard English?

Reviewer #1: Yes

**********

Reviewer #1: Thank you very much for the opportunity to review this paper, which is both very detailed and well written.

I have only very few comments I believe to be addressworthy.

Overall:

You are focusing almost exclusively on the occupational hazards but sterilization efficacy and not increasing the risk for recontamination are also important. Could you add a comment on whether you believe this setup might increase the recontamination risk, e.g. through the additional airflow?

Sterilization efficacy often has to be validated (and routinely checked). Regarding the guidelines applicable to your institution, would the addition of the exhaust system require a revalidation of the machines?

Does loading/unloading the disinfector change the expected temperature and/or particle distribution?

Abstract:

Please write the “p” in “p-value” in lower case.

Introduction:

Line 74 Considering the broad readership of PONE, it might be helpful to give a short description of the definition of the A0 Value (equivalent time (in seconds) at a reference temperature of 80 °C required to achieve the same microbial inactivation as the actual thermal process).

Lines 126 – 129 Considering the steam comes from a disinfector, why do you assume it to increase the “risk of environmental and personnel infection”? Could you provide literature evaluating the infection risk from disinfectors?

Methods:

If you add a sentence to your experimental design emphasizing that you did not involve human participants, the supplementary file stating no humans were involved might not be necessary.

Lines 236 – 242 If manual opening leaves the data unreliable, is there still an element of compliance involved, even with the exhaust, i.e. is there still a risk for people opening the disinfector rapidly?

Text for Fig 4: Please explicitly explain numbers 1 and 2 shown in the image in the image description

Line 256 Please add the manufacturer of the thermometer.

Line 258 Please add the manufacturer of the particle counter.

Line 280 Please write the “p” in “p-value” in lower case.

Results:

I would suggest adding the data table to the main manuscript and publish the whole document without supplementary information. This would increase accessibility, and it does not seem to be too much data.

**********

what does this mean?). If published, this will include your full peer review and any attached files.

If you choose “no”, your identity will remain anonymous but your review may still be made public.

**Do you want your identity to be public for this peer review?**  For information about this choice, including consent withdrawal, please see our Privacy Policy

Reviewer #1: No

**********

[NOTE: If reviewer comments were submitted as an attachment file, they will be attached to this email and accessible via the submission site. Please log into your account, locate the manuscript record, and check for the action link "View Attachments". If this link does not appear, there are no attachment files.]

To ensure your figures meet our technical requirements, please review our figure guidelines: https://journals.plos.org/plosone/s/figures

You may also use PLOS’s free figure tool, NAAS, to help you prepare publication quality figures: https://journals.plos.org/plosone/s/figures#loc-tools-for-figure-preparation.

NAAS will assess whether your figures meet our technical requirements by comparing each figure against our figure specifications.

# Author comment: Author response to Decision Letter 1

**DOI:** 10.1371/journal.pone.0360909.r002

10 Aug 2026

We sincerely appreciate the constructive comments raised by the academic editor and reviewers. Revisions and supplements have been made to the manuscript as suggested. Detailed point-by-point responses are provided below.

Response to academic Editor

Comment 1. Insufficient description and regulatory context of the "boiling disinfector"

Response: To address the inadequate description of equipment information and regulatory background of boiling disinfectors, the following revisions have been implemented:

1. Supplementary information on equipment classification and market compliance. In the Introduction section, we clarify that boiling disinfectors fall under Class II medical devices in China, complying with the industrial standard YY/T 1687-2019. Each device is delivered with complete manufacturer Instructions for Use (IFU) that specify standardized validated disinfection parameters. Full descriptions of the statutory classification, intended clinical applications, and market access requirements of the equipment are provided.

2. Improved framework of mandatory equipment qualification. Combined with ISO 15883 and YY/T 1687 standards, we elaborate on three mandatory qualification procedures (IQ, OQ, PQ) that all medical moist heat disinfection equipment must complete before clinical deployment, illustrate the inspection scope of each qualification stage, and supplement comprehensive content regarding standardized quality control of the equipment.

3. Supplementary calibration qualification of the tested unit. The boiling disinfector used in all tests passed full IQ/OQ/PQ validation. All built-in temperature sensors, heating assemblies and timing controllers received annual metrological calibration in accordance with manufacturer IFU and YY/T 1687-2019. The equipment operated under fully compliant conditions throughout the experiment, eliminating data bias caused by equipment drift or parameter deviation.

4. Demonstration that ICES retrofitting does not invalidate original equipment qualification status. A standalone paragraph has been added to the Discussion section to illustrate that ICES is an external non-destructive auxiliary exhaust assembly. Only adhesive-mounted magnets and Hall sensors are installed without drilling, disassembly or modification of original wiring. No alterations are made to the chamber structure, heating system, temperature control program, water pipeline, door sealing or other core process modules, so the original IQ/OQ/PQ validated status of the boiling disinfector remains intact.

5. Clarification of the root source of occupational risks. The Discussion section states that steam overflow during hatch opening is not induced by defective equipment, improper installation or non-compliant operations. Instead, it represents an inherent physical design limitation of front-flip hatch-type boiling disinfectors. This clear differentiation of risk origins excludes insufficient equipment quality control as a contributing factor.

Comment 2. Clarification of disinfection versus sterilization principles

Response:

1. Distinct differentiation between moist heat disinfection and moist heat sterilization. In the Introduction section, we distinguish the two thermal processing modalities across four dimensions (heat transfer medium, microbial inactivation capacity, sterility assurance level, and clinical application) based on two international standards: ISO 15883 and ISO 17665.

2. Unified supporting standards for equipment processing. YY/T 1687-2019 is specified as the exclusive industrial standard for boiling disinfectors throughout the manuscript. The A₀ value evaluation criterion is cross-referenced with ISO 15883 and the domestic CSSD standard WS 310.3, clearly defining standardized disinfection temperatures, holding durations and benchmarks for microbial inactivation.

3. Elaborated the important clinical application scenarios of boiling disinfectors. We expand descriptions of the clinical demand for precision micro-instruments and lumened devices, explain the limitations of automated spray washer-disinfectors including instrument loss and unreachable cleaning dead zones, fully illustrate the clinical necessity of boiling disinfectors, and clarify their applicable scenarios.

Comment 3. Lack of standards-based discussion

Response: To remedy the insufficient standard-related discussion in the original manuscript, we have integrated multiple sets of standards covering moist heat disinfection, medical device quality control, risk management and equipment qualification throughout the text:

1. Standards for moist heat disinfection and equipment qualification. Frequent citations of ISO 15883, YY/T 1687 and WS 310.3 are incorporated to fully interpret the mandatory industrial requirements for A₀ value assessment and IQ/OQ/PQ validation of thermal disinfection equipment.

2. Medical device risk management standards. ISO 14971 risk management guidelines are cited in the root-cause risk analysis paragraph. Following the risk control hierarchy of "engineering control priority", we demonstrate that the external ICES exhaust assembly constitutes a compliant engineering intervention to mitigate inherent occupational hazards of the equipment, filling the gap in risk management standard discussion.

3. Verification of ICES compliance with equipment regulatory requirements. The manuscript clarifies that ICES is a non-intrusive external add-on solution without destructive modification or adjustment of core process parameters of the boiling disinfector, which will not compromise the original validated equipment status and conforms to regulatory specifications governing medical device modification risk control.

Comment 4. Root cause analysis of occupational risk is missing

Response: To address the absence of comprehensive root-cause analysis for occupational exposure hazards, a full analytical paragraph has been added at the start of the Discussion section to decompose risk contributors layer by layer:

1. The tested boiling disinfector completed full IQ/OQ/PQ validation. Its hardware configuration, installation layout and routine maintenance fully comply with manufacturer IFU and industrial standards, ruling out inadequate equipment performance, defective installation or incomplete quality control as risk drivers.

2. All experimental operations strictly followed standardized manufacturer protocols with standardized operator movements, eliminating excessive steam overflow caused by non-compliant manual handling.

3. Instant massive steam surge upon hatch opening is an objective physical phenomenon arising from positive pressure accumulation within the sealed heated chamber. The front-flip hatch design forces technicians to stand in close proximity to high-temperature steam plumes, which cannot be eliminated by standardized disinfection cycles or basic room ventilation alone.

4. In line with ISO 14971 risk management logic, steam overflow hazards are inherent structural limitations of the equipment as manufactured. Personal protective equipment and procedural optimization cannot fully eliminate such risks, which makes external engineering control devices such as ICES a targeted and necessary risk mitigation strategy.

Comment 5. Limited verification of effectiveness

Response: We objectively acknowledge the experimental limitations and supplement engineering principles and short-term stability data to strengthen argumentation:

1. We acknowledge the limitations of this study. All validation tests were performed on only one hatch-type boiling disinfector. The limited sample size restricts the generalizability of the research conclusions to a certain extent. In follow-up studies, we will incorporate additional models of equipment for testing to enrich the experimental dataset. The ICES features a simple structure and operating principle. It realizes protective function via magnetic induction hatch detection and instantaneous negative-pressure steam extraction and requires no customized modifications for specific equipment models. Its modular external installation combined with flexibly bendable pipelines enables compatibility with devices of various shapes. Based on the design characteristics of the device, we anticipate that this system is applicable to most commercially available hatch-type boiling disinfectors.

2. All effectiveness verification tests were carried out under no-load conditions, for two objective reasons. Firstly, all precision micro-instruments and lumened devices at the testing hospital were reserved for standby use in operating theatres during the trial period, and no spare instruments were available for experimental loading. Secondly, all medical devices are fully submerged under the water surface throughout the boiling disinfection cycle; saturated steam accumulates in the headspace above the liquid level, and there is no remarkable difference in chamber temperature or total steam volume generated during operation regardless of whether instrument loads are placed inside the chamber.

3. Supplementary data on continuous operational reliability. Four months of continuous on-site clinical operation data are added to the Discussion section. The ICES prototype ran daily alongside routine boiling disinfection workflows, with zero failures of the centrifugal fan, Hall sensor and delay control modules, no attenuation of sensing sensitivity, and no circuit short-circuiting or pipeline deformation observed, which preliminarily verifies its short-term operational stability.

4. ICES exerts no adverse influence on the inherent performance of the host boiling disinfector, with a standalone explanatory paragraph added to the Discussion. ICES adopts a non-destructive external installation scheme that leaves all core performance metrics of the disinfector unchanged, including disinfection cycle duration, internal chamber temperature and A₀ value. This retrofitting solution does not raise additional maintenance costs, nor will it invalidate the original Installation Qualification, Operational Qualification and Performance Qualification status of the host equipment.

Comment 6. PLOS ONE reproducibility requirements

Response: To meet the journal’s reproducibility criteria, we supplemented standardized information regarding equipment, measuring instruments and retrofitting solutions to enable independent replication of the entire experiment:

1. Complete specifications and qualification records of the boiling disinfector. Full documentation of the tested unit’s brand, external dimensions, chamber volume and standard working water volume is retained in the manuscript. Supplementary text confirms full IQ/OQ/PQ validation and annual metrological calibration of all core components, with all experimental workflows conducted in strict accordance with ISO 15883 to ensure consistent, repeatable test conditions.

2. Supplementary calibration information for measuring instruments. We specify that both the dual-channel thermometer and airborne particle counter were sourced from certified manufacturers and calibrated by accredited metrological institutions to guarantee repeatable and comparable detection data.

3. Comprehensive hardware parameter documentation for ICES. The manuscript retains complete dimensional, material and electrical specifications of all components including the centrifugal fan, Hall effect switch, relays and piping, providing full hardware references for independent replication. The selected centrifugal fan provides sufficient airflow redundancy. Minor fluctuations in flow rate will not impair steam extraction efficiency. This study focuses on on-site performance verification rather than computational fluid dynamics simulation. Therefore, the supplied hardware parameters are adequate to replicate the system, and supplementary fan-sizing calculation is beyond the scope of the present work.

4. Enhanced risk assessment for equipment modification. The manuscript fully analyzes the non-destructive external installation, complete electrical isolation and zero alteration of core equipment processes of ICES.

Response to Reviewer

Comment 1:You are focusing almost exclusively on the occupational hazards but sterilization efficacy and not increasing the risk for recontamination are also important. Could you add a comment on whether you believe this setup might increase the recontamination risk, e.g. through the additional airflow? Sterilization efficacy often has to be validated (and routinely checked). Regarding the guidelines applicable to your institution, would the addition of the exhaust system require a revalidation of the machines? Does loading/unloading the disinfector change the expected temperature and/or particle distribution?

Response: First, the ICES serves as an independent external auxiliary device. Only a magnet and a Hall sensor are adhesively attached to the boiling disinfector, without any modifications to the disinfector’s chamber, temperature control system, water pipeline or sealing structure. Its installation does not alter any critical process parameters validated via IQ/OQ/PQ, and relevant discussions have been provided in the manuscript. In accordance with the Chinese standard YY/T 1687 and the hospital’s equipment management specifications, a full revalidation of the host disinfection equipment is not required for such an independent external auxiliary protection device.

Second, the system does not elevate risks of environmental contamination or recontamination of instruments. The core components of ICES include a stainless steel centrifugal fan and corrosion-resistant exhaust pipelines with smooth surfaces that resist microbial adhesion. The extracted steam is directly discharged outdoors through the building’s centralized exhaust duct system and will not aggravate contamination within the CSSD workspace.

Finally, instrument loading and unloading operations cannot change the temperature and aerosol distribution associated with instantaneous steam release upon hatch opening. As illustrated in the experimental design section of the manuscript, all instruments remain fully submerged under the water surface throughout boiling disinfection. Submerged instrument loads exert minimal influence on the total volume of saturated steam accumulated in the headspace above the liquid level.

Comment 2:Abstract: Please write the “p” in “p-value” in lower case.

Response: Following the reviewer’s suggestion, "P-value" in the Abstract has been revised to the lowercase form "p-value".

Comment 3: Introduction: Line 74 Considering the broad readership of PONE, it might be helpful to give a short description of the definition of the A0 Value (equivalent time (in seconds) at a reference temperature of 80 °C required to achieve the same microbial inactivation as the actual thermal process).

Response: We have supplemented the complete definition of the A₀ value at the corresponding position in the Introduction, consistent with standard formal definitions.

Comment 4: Lines 126 – 129 Considering the steam comes from a disinfector, why do you assume it to increase the “risk of environmental and personnel infection”? Could you provide literature evaluating the infection risk from disinfectors?

Response: Steam generated during boiling disinfection itself poses low infection risk. However, the decontamination area of the CSSD is classified as a contaminated zone. Massive hot steam surges outward immediately after hatch opening, disturbing pre-existing bioaerosols in the environment, facilitating aerosol suspension and uncontrolled dispersion in ambient air, and thereby elevating exposure risks for staff. Relevant literature citations have been added to this paragraph of the manuscript to support this viewpoint.

Comment 5: Methods: If you add a sentence to your experimental design emphasizing that you did not involve human participants, the supplementary file stating no humans were involved might not be necessary.

Response: We have added a statement in the manuscript clarifying that this trial involved no experiments on human participants and recruited no human subjects. Meanwhile, in compliance with PLOS ONE submission requirements, we retain the relevant declaration regarding the absence of human participants within the supplementary file to prevent format-related issues during manuscript evaluation.

Comment 6: Lines 236 – 242 If manual opening leaves the data unreliable, is there still an element of compliance involved, even with the exhaust, i.e. is there still a risk for people opening the disinfector rapidly?

Response: A distinction should be drawn between standardized experimental test conditions and routine clinical application scenarios. The self-made uniform-speed hatch-opening auxiliary device described in the manuscript was adopted solely to eliminate confounding variables induced by inconsistent manual hatch-opening speeds and ensure comparability of experimental datasets across groups; it was used exclusively for quantitative performance testing of the ICES.

When the ICES system is deployed in real clinical settings, this auxiliary device is not required. Regardless of variations in operators’ hatch-opening speed, the Hall sensor activates the exhaust fan immediately upon initial hatch opening to extract steam, thereby mitigating massive steam overflow and reducing risks of occupational exposure.

Comment 7: Text for Fig 4: Please explicitly explain numbers 1 and 2 shown in the image in the image description. Line 256 Please add the manufacturer of the thermometer. Line 258 Please add the manufacturer of the particle counter. Line 280 Please write the “p” in “p-value” in lower case.

Response: All revisions have been completed. Labels 1 and 2 in Figure 4 are explicitly defined within the figure legend. Manufacturer information for the dual-channel thermometer and airborne particle counter has been supplemented in the specified lines. All instances of "P-value" throughout the manuscript have been corrected to the lowercase form "p-value".

Comment 8: Results: I would suggest adding the data table to the main manuscript and publish the whole document without supplementary information. This would increase accessibility, and it does not seem to be too much data.

Response: After comprehensive consideration of manuscript length and layout of figures and tables, we prefer to keep the data tables as supplementary information. All supplementary materials are openly accessible without impairing data availability. The final formatting arrangement will follow subsequent instructions from the journal editors.

Attachment Submitted filename: Response to Reviewers.docx

# Peer review: Decision Letter 1

**DOI:** 10.1371/journal.pone.0360909.r003

**Authors:** Paulo Laranjeira

8 Sep 2026

Dear Dr. Zheng,

Thank you for submitting your manuscript to PLOS One. After careful consideration, we feel that it has merit but does not fully meet PLOS One’s publication criteria as it currently stands. Therefore, we invite you to submit a revised version of the manuscript that addresses the points raised during the review process.

Please submit your revised manuscript by Nov 07 2026 11:59PM. If you will need more time than this to complete your revisions, please reply to this message or contact the journal office at plosone@plos.org. When you're ready to submit your revision, log on to https://www.editorialmanager.com/pone/ and select the 'Submissions Needing Revision' folder to locate your manuscript file.

- A letter that responds to each point raised by the academic editor and reviewer(s). You should upload this letter as a separate file labeled 'Response to Reviewers'.
- A marked-up copy of your manuscript that highlights changes made to the original version. You should upload this as a separate file labeled 'Revised Manuscript with Track Changes'.
- An unmarked version of your revised paper without tracked changes. You should upload this as a separate file labeled 'Manuscript'.

If you would like to make changes to your financial disclosure, please include your updated statement in your cover letter. Guidelines for resubmitting your figure files are available below the reviewer comments at the end of this letter.

If applicable, we recommend that you deposit your laboratory protocols in protocols.io to enhance the reproducibility of your results. Protocols.io assigns your protocol its own identifier (DOI) so that it can be cited independently in the future. For instructions see: https://journals.plos.org/plosone/s/submission-guidelines#loc-laboratory-protocols. Additionally, PLOS One offers an option for publishing peer-reviewed Lab Protocol articles, which describe protocols hosted on protocols.io. Read more information on sharing protocols at https://plos.org/protocols?utm_medium=editorial-email&utm_source=authorletters&utm_campaign=protocols.

As the corresponding author, your ORCID iD is verified in the submission system and will appear in the published article. PLOS supports the use of ORCID, and we encourage all coauthors to register for an ORCID iD and use it as well. Please encourage your coauthors to verify their ORCID iD within the submission system before final acceptance, as unverified ORCID iDs will not appear in the published article. *Only*  the individual author can complete the verification step; PLOS staff *cannot*  verify ORCID iDs on behalf of authors.

We look forward to receiving your revised manuscript.

Kind regards,

Paulo Laranjeira

Academic Editor

PLOS One

Journal Requirements:

If the reviewer comments include a recommendation to cite specific previously published works, please review and evaluate these publications to determine whether they are relevant and should be cited. There is no requirement to cite these works unless the editor has indicated otherwise.

[Note: HTML markup is below. Please do not edit.]

Reviewers' comments:

Reviewer’s Responses to Questions

**Comments to the Author**

Reviewer #2: All comments have been addressed

Reviewer #3: (No Response)

**********

2. Is the manuscript technically sound, and do the data support the conclusions?

Reviewer #2: Partly

Reviewer #3: Yes

**********

3. Has the statistical analysis been performed appropriately and rigorously?

Reviewer #2: Yes

Reviewer #3: Yes

**********

4. Have the authors made all data underlying the findings in their manuscript fully available?

The PLOS Data policy

Reviewer #2: Yes

Reviewer #3: Yes

**********

5. Is the manuscript presented in an intelligible fashion and written in standard English?

Reviewer #2: No

Reviewer #3: Yes

**********

Reviewer #2: The title appears a little convoluted. It could be more precise and clearer.

2. Provide the achieved results in the form of percentages in the last section of your abstract.

3. Provide the key contribution of your work in the form of a Research Question (RQ).

4. The abstract must be reorganized to clearly state the purpose, methodology, and key quantitative results. The introduction needs a logical flow that connects background, problem, gap, and motivation. The authors should follow the below papers and improve it accordingly.

i. Advanced federated ensemble internet of learning approach for cloud based medical healthcare monitoring system

ii. High-precision brain tumor classification from MRI images using an advanced hybrid deep learning method with minimal radiation exposure

iii. Deep learning-based multi-class classification of thyroid disorders on Tc-99m scintigraphy using modified DenseNet-201

5. Don’t use abbreviations in the keywords.

6. At the end of the related works section provide a paragraph that should encapsulate on the knowledge that what shortcomings has motivated you to do this specific work.

7. Some abbreviations are not defined for their first appearance.

8. There are a lot of grammatical and typos; the authors are suggested to do a strong proofread before the revision submission and remove them.

9. Add a time-series measurement (e.g., temperature vs. time graphs for the first 30 seconds post-opening) to show if the ICES also shortens the exposure window.

10. State clearly if the machine was tested multiple times on the same day or across days to account for environmental variables (e.g., ambient room temperature, humidity). If not randomized, add this as a limitation.

11. While not mandatory, adding a measurement of relative humidity or air velocity at T2 would provide a more complete risk assessment (thermal discomfort is a combination of heat and humidity).

Reviewer #3: I have reviewed the revised manuscript, which presents a practical interlocked centrifugal exhaust system for hatch‑type boiling disinfectors and addresses a genuine occupational safety issue. The authors have responded thoroughly to previous criticisms, adding regulatory context, standards, and hardware details, so the scientific foundation is now solid. However, two minor points still need attention.

First, the experimental limitations (testing on only one machine and under no‑load conditions) are disclosed only in the final Limitations section, while the Results and the opening of the Discussion use strong, generalised phrasing. I recommend stating these constraints explicitly in the Methods, for instance by adding a sentence that this is a preliminary single‑unit no‑load validation, and slightly moderating the wording in the Discussion to reflect the preliminary scope.

Second, the fan noise (80 dB) and condensate residue are mentioned but no plans or feasibility assessments are provided. I suggest adding brief comments: for noise, note that the ambient CSSD noise is typically 60‑65 dB and that operator comfort should be evaluated; for condensate, mention that a drainage port has been bench‑tested and will be included in future iterations. The authors’ previous revisions were exemplary, and these remaining adjustments are straightforward. I therefore recommend acceptance after minor revision.

**********

what does this mean?). If published, this will include your full peer review and any attached files.

If you choose “no”, your identity will remain anonymous but your review may still be made public.

**Do you want your identity to be public for this peer review?**  For information about this choice, including consent withdrawal, please see our Privacy Policy

Reviewer #2: No

Reviewer #3: No

**********

[NOTE: If reviewer comments were submitted as an attachment file, they will be attached to this email and accessible via the submission site. Please log into your account, locate the manuscript record, and check for the action link "View Attachments". If this link does not appear, there are no attachment files.]

To ensure your figures meet our technical requirements, please review our figure guidelines: https://journals.plos.org/plosone/s/figures

You may also use PLOS’s free figure tool, NAAS, to help you prepare publication quality figures: https://journals.plos.org/plosone/s/figures#loc-tools-for-figure-preparation.

NAAS will assess whether your figures meet our technical requirements by comparing each figure against our figure specifications.

# Author comment: Author response to Decision Letter 2

**DOI:** 10.1371/journal.pone.0360909.r004

12 Sep 2026

We sincerely appreciate the reviewers’ constructive comments and thoughtful suggestions, which have substantially helped us improve the quality and clarity of this manuscript. We have carefully addressed each comment point‑by‑point as below.

Reviewer #2:

Comment 1. The title appears a little convoluted. It could be more precise and clearer.

Response: After careful evaluation, we prefer to retain the original title: Design and verification of an interlocked centrifugal exhaust system for hatch‑type boiling disinfectors. This title comprehensively covers all core elements of the present study, including the research activities (design and verification), the full name of the proposed interlocked centrifugal exhaust system (ICES), and the target equipment (hatch‑type boiling disinfectors). No ambiguous wording is used. All essential metadata required for literature indexing and database retrieval are included. Therefore, we keep the original title unchanged in the revised manuscript.

Comment 2. Provide the achieved results in the form of percentages in the last section of your abstract.

Response: We prepared the abstract following common writing practices for biomedical‑engineering research manuscripts. Our abstract consists of four distinct sections: objective, methods, results, and conclusion. All quantitative percentage‑based efficacy outcomes of ICES are fully and explicitly presented within the Results section of the Abstract. The Conclusion section is designed to interpret the practical implications and application value drawn from our experimental findings, instead of restating detailed numerical results, which would create unnecessary textual redundancy. Readers can retrieve all key percentage‑reduction data from the Results section of the Abstract. For these reasons, we keep the original wording of the Abstract Conclusion unchanged.

Comment 3. Provide the key contribution of your work in the form of a Research Question (RQ).

Response: Thank you for this suggestion. Following the narrative logic of the three recommended reference papers, we have re‑phrased the final paragraph of the Introduction. A research question (RQ) is explicitly stated at the beginning of this paragraph, which is immediately followed by the key outcomes and contributions of the present study. No new substantive content has been added; all information is inherited from the original manuscript, with only sentence structure and expression revised. This revision can be found in the final paragraph of the Introduction in the revised manuscript.

Comment 4. The abstract must be reorganized to clearly state the purpose, methodology, and key quantitative results. The introduction needs a logical flow that connects background, problem, gap, and motivation. The authors should follow the below papers and improve it accordingly.

i. Advanced federated ensemble internet of learning approach for cloud based medical healthcare monitoring system

ii. High-precision brain tumor classification from MRI images using an advanced hybrid deep learning method with minimal radiation exposure

iii. Deep learning-based multi-class classification of thyroid disorders on Tc-99m scintigraphy using modified DenseNet-201

Response: Thank you for this constructive suggestion and for providing the three reference papers. We have learned from their explicit narrative framework, and relevant adjustments have already been implemented for the formulation of the research question and study contributions as described in Comment 3.

For the abstract, the current version is structured sequentially with objective, methods, key quantitative results and conclusion sections, which systematically reports the research purpose of mitigating occupational exposure risks of hatch type boiling disinfectors, the configuration of the interlocked centrifugal exhaust system (ICES), experimental measurement indicators, statistical approaches, core quantitative reduction data of temperature and airborne particle counts, as well as the practical significance of this work. All required information elements are already fully covered within the existing abstract, and no further reorganization of the abstract structure is performed.

For the Introduction, the existing text follows the logical chain of background clinical problem research gap research motivation. It begins with the routine disinfection modalities adopted in the central sterile supply department, elaborates the widespread application scenario of moist heat disinfection, explains the indispensable supplementary role of boiling disinfectors for special complex instruments, describes the operating workflow of hatch type boiling disinfectors, and further illustrates the occupational exposure hazards induced by instantaneous steam overflow during top cover opening. Subsequently, the limitations of currently available protective strategies and patented technical solutions are comprehensively discussed to clarify the existing research gap. Finally, the research motivation of developing the ICES is presented. This complete logical progression meets the narrative requirement raised by you. Combined with the revision in Comment 3, an explicit research question together with key study outcomes has been added at the end of the Introduction to further strengthen the logical closure of this section. Considering that the core narrative and argumentation are already complete, we retain the original organizational structure of the Introduction without additional large scale rearrangement.

Comment 5. Don’t use abbreviations in the keywords.

Response: We have double checked our keywords. The keyword list was submitted via the journal submission system rather than being embedded within the main manuscript file. The submitted keywords are as follows: Central sterile supply department; Boiling disinfector; Occupational protection. All keywords are written in complete full length terms without any abbreviations.

Comment 6. At the end of the related works section provide a paragraph that should encapsulate on the knowledge that what shortcomings has motivated you to do this specific work.

Response: We understand that you are asking for a concise encapsulation of the shortcomings of existing approaches that motivated the present research. This content is fully presented in the penultimate paragraph of the Introduction. This paragraph systematically summarizes the limitations of passive protective measures and existing patented technical solutions for mitigating steam‑overflow‑related occupational hazards, and clarifies why these existing strategies cannot resolve the clinical challenges of hatch‑type boiling disinfectors. This paragraph serves exactly the summarizing function you requested. Adding another standalone summary paragraph would introduce redundant repetitive information without bringing new insights. Accordingly, we retain the original text of this paragraph without further revision. Together with the newly added research question and key outcomes in the final paragraph of the Introduction (see Comment 3), the logical chain from identified shortcomings to research motivation is complete.

Comment 7. Some abbreviations are not defined for their first appearance.

Response: We have systematically re examined every acronym and symbol throughout the abstract and full text manuscript. We have verified that each abbreviation is accompanied by its complete full name upon its first occurrence, including ICES, CSSD, ISO, SAL, IQ, OQ, PQ, PVC, ABS and A₀. Symbols T1 and T2 are also clearly defined in the Methods subsection of the Abstract before they are used in the Results subsection. No undefined abbreviations or symbols remain in the revised manuscript.

Comment 8. There are a lot of grammatical and typos; the authors are suggested to do a strong proofread before the revision submission and remove them.

Response: We have conducted comprehensive proof‑reading across the entire manuscript. Several typographical mistakes, duplicated words and awkward grammatical expressions were identified and corrected, including the duplicated definite article “the” and other minor wording imperfections.

Comment 9. Add a time-series measurement (e.g., temperature vs. time graphs for the first 30 seconds post-opening) to show if the ICES also shortens the exposure window.

Response: We greatly appreciate this suggestion. Supplementary experiments were carried out to record temperature variations at the two measurement sites with and without the ICES. The two positions were the chamber edge (T1) and the technician’s facial position (T2). Time-series plots were generated based on the acquired data.

Preliminary tests revealed that temperatures at both sampling positions returned to baseline levels within 15 seconds after opening the top cover, regardless of whether the ICES was activated. For this reason, temperature monitoring was terminated at 15 s, and we did not perform measurements covering the 30 s after top cover opening.

The time-series plots clearly demonstrate that the occupational injury risk induced by high-temperature steam is substantially reduced when the ICES is activated. Specifically, after ICES deployment, temperatures at both positions show nearly no obvious rise upon opening the top cover, and the time window for steam exposure is almost eliminated. The new experimental data and figures have been integrated into the revised manuscript.

Comment 10. State clearly if the machine was tested multiple times on the same day or across days to account for environmental variables (e.g., ambient room temperature, humidity). If not randomized, add this as a limitation.

Response: All experiments were conducted across three non consecutive days. The test time points were not randomly selected. However, the environmental temperature and humidity within the central sterile supply department are maintained in a relatively stable and controlled range. In addition, the test equipment, measuring instruments, operators and objective test parameters remained consistent throughout all trials. Given these stable conditions, we consider the impact of non randomized test timing negligible. Therefore, we do not include this point in the Limitations section.

Comment 11. While not mandatory, adding a measurement of relative humidity or air velocity at T2 would provide a more complete risk assessment (thermal discomfort is a combination of heat and humidity).

Response: We apologize that local relative humidity and air velocity at T1 and T2 were not recorded during the experiments. As described in our response to Comment 10, the central sterile supply department is an enclosed space with strictly controlled temperature and humidity. Variations in local humidity and air velocity were minimal under these stable indoor conditions. In addition, high sensitivity instruments for point measurement of these parameters were unavailable, and on site measurement at these locations presented practical operational challenges. We believe that the absence of these two measurements does not affect the core conclusions of this study.

Reviewer #3:

First, the experimental limitations (testing on only one machine and under no load conditions) are disclosed only in the final Limitations section, while the Results and the opening of the Discussion use strong, generalised phrasing. I recommend stating these constraints explicitly in the Methods, for instance by adding a sentence that this is a preliminary single unit no load validation, and slightly moderating the wording in the Discussion to reflect the preliminary scope.

Response: We have added a sentence in the Methods section to state that this validation is a preliminary single-unit prototype test. In addition, we have revised the heading of the first Discussion subsection and moderated the opening statements of Discussion to clearly reflect the preliminary and limited scope of the present experiment, avoiding overgeneralized conclusions.

Second, the fan noise (80 dB) and condensate residue are mentioned but no plans or feasibility assessments are provided. I suggest adding brief comments: for noise, note that the ambient CSSD noise is typically 60 65 dB and that operator comfort should be evaluated; for condensate, mention that a drainage port has been bench tested and will be included in future iterations. The authors’ previous revisions were exemplary, and these remaining adjustments are straightforward. I therefore recommend acceptance after minor revision.

Response: We appreciate this thoughtful suggestion. We have supplemented relevant discussions in the Limitations section. For fan noise, we added background CSSD ambient noise levels (60–65 dB) and noted that fan operation lasts less than one minute per use, which limits high-noise exposure. We also added that operator comfort assessment will be required in future field evaluations. For condensate residue, we added that a 4 mm drainage port with a plastic tube has completed bench testing with effective drainage performance, and this solution will be implemented in future prototype iterations.

Attachment Submitted filename: Response_to_Reviewers_auresp_2.docx

# Peer review: Decision Letter 2

**DOI:** 10.1371/journal.pone.0360909.r005

**Authors:** Paulo Laranjeira

5 Oct 2026

Design and verification of an interlocked centrifugal exhaust system for hatch-type boiling disinfectors

PONE-D-26-23588R2

Dear Dr. Zheng,

We’re pleased to inform you that your manuscript has been judged scientifically suitable for publication and will be formally accepted for publication once it meets all outstanding technical requirements.

Within one week, you’ll receive an e-mail detailing the required amendments. When these have been addressed, you’ll receive a formal acceptance letter and your manuscript will be scheduled for publication.

An invoice will be generated when your article is formally accepted. Please note, if your institution has a publishing partnership with PLOS and your article meets the relevant criteria, all or part of your publication costs will be covered. Please make sure your user information is up-to-date by logging into Editorial Manager at Editorial Manager® and clicking the ‘Update My Information' link at the top of the page. For questions related to billing, please contact billing support.

If your institution or institutions have a press office, please notify them about your upcoming paper to help maximize its impact. If they’ll be preparing press materials, please inform our press team as soon as possible -- no later than 48 hours after receiving the formal acceptance. Your manuscript will remain under strict press embargo until 2 pm Eastern Time on the date of publication. For more information, please contact onepress@plos.org.

Kind regards,

Paulo Laranjeira

Academic Editor

PLOS One

Additional Editor Comments (optional):

Reviewers' comments:

Reviewer’s Responses to Questions

**Comments to the Author**

Reviewer #2: All comments have been addressed

**********

2. Is the manuscript technically sound, and do the data support the conclusions?

Reviewer #2: Yes

**********

3. Has the statistical analysis been performed appropriately and rigorously?

Reviewer #2: Yes

**********

4. Have the authors made all data underlying the findings in their manuscript fully available?

The PLOS Data policy

Reviewer #2: Yes

**********

5. Is the manuscript presented in an intelligible fashion and written in standard English?

Reviewer #2: Yes

**********

Reviewer #2: The authors have improved their paper after the revision and now the paper is acceptable for publication.

**********

what does this mean?). If published, this will include your full peer review and any attached files.

If you choose “no”, your identity will remain anonymous but your review may still be made public.

**Do you want your identity to be public for this peer review?**  For information about this choice, including consent withdrawal, please see our Privacy Policy

Reviewer #2: No

**********

# Editor report: Acceptance letter

**DOI:** 10.1371/journal.pone.0360909.r006

**Authors:** Paulo Laranjeira

PONE-D-26-23588R2

PLOS One

Dear Dr. Zheng,

I'm pleased to inform you that your manuscript has been deemed suitable for publication in PLOS One. Congratulations! Your manuscript is now being handed over to our production team.

At this stage, our production department will prepare your paper for publication. This includes ensuring the following:

* All references, tables, and figures are properly cited

* All relevant supporting information is included in the manuscript submission,

* There are no issues that prevent the paper from being properly typeset

You will receive further instructions from the production team, including instructions on how to review your proof when it is ready. Please keep in mind that we are working through a large volume of accepted articles, so please give us a few days to review your paper and let you know the next and final steps.

Lastly, if your institution or institutions have a press office, please let them know about your upcoming paper now to help maximize its impact. If they'll be preparing press materials, please inform our press team within the next 48 hours. Your manuscript will remain under strict press embargo until 2 pm Eastern Time on the date of publication. For more information, please contact onepress@plos.org.

You will receive an invoice from PLOS for your publication fee after your manuscript has reached the completed accept phase. If you receive an email requesting payment before acceptance or for any other service, this may be a phishing scheme. Learn how to identify phishing emails and protect your accounts at https://explore.plos.org/phishing.

If we can help with anything else, please email us at customercare@plos.org.

Thank you for submitting your work to PLOS One and supporting open access.

Kind regards,

PLOS One Editorial Office Staff

on behalf of

Dr. Paulo Laranjeira

Academic Editor

PLOS One
